# Transcriptome analysis of *Phelipanche aegyptiaca* seed germination mechanisms stimulated by fluridone, TIS108, and GR24

**DOI:** 10.1371/journal.pone.0187539

**Published:** 2017-11-03

**Authors:** Ya Zhou Bao, Zhao Qun Yao, Xiao Lei Cao, Jin Feng Peng, Ying Xu, Mei Xiu Chen, Si Feng Zhao

**Affiliations:** Key Laboratory at Universities of Xinjiang Uygur Autonomous Region for Oasis Agricultural Pest Management and Plant Protection Resource Utilization, Shihezi University, Shihezi, China; Youngstown State University, UNITED STATES

## Abstract

*P*. *aegyptiaca* is one of the most destructive root parasitic plants worldwide, causing serious damage to many crop species. Under natural conditions *P*. *aegyptiaca* seeds must be conditioned and then stimulated by host root exudates before germinating. However, preliminary experiments indicated that TIS108 (a triazole-type inhibitor of strigolactone) and fluridone (FL, an inhibitor of carotenoid-biosynthesis) both stimulated the germination of *P*. *aegyptiaca* seeds without a water preconditioning step (i.e. unconditioned seeds). The objective of this study was to use deep RNA sequencing to learn more about the mechanisms by which TIS108 and FL stimulate the germination of unconditioned *P*. *aegyptiaca* seeds. Deep RNA sequencing was performed to compare the mechanisms of germination in the following treatments: (i) unconditioned *P*. *aegyptiaca* seeds with no other treatment, (ii) unconditioned seeds treated with 100 mg/L TIS108, (iii) unconditioned seeds treated with 100 mg/L FL + 100 mg/L GA_3_, (iv) conditioned seeds treated with sterile water, and (v) conditioned seeds treated with 0.03 mg/L GR24. The *de novo* assembled transcriptome was used to analyze transcriptional dynamics during seed germination. The key gene categories involved in germination were also identified. The results showed that only 119 differentially expressed genes were identified in the conditioned treatment vs TIS108 treatment. This indicated that the vast majority of conditions for germination were met during the conditioning stage. Abscisic acid (ABA) and gibberellic acid (GA) played important roles during *P*. *aegyptiaca* germination. The common pathway of TIS108, FL+GA_3_, and GR24 in stimulating *P*. *aegyptiaca* germination was the simultaneous reduction in ABA concentrations and increase GA concentrations. These results could potentially aid the identification of more compounds that are capable of stimulating *P*. *aegyptiaca* germination. Some potential target sites of TIS108 were also identified in our transcriptome data. The results of this experiment suggest that TIS108 and FL+GA_3_ could be used to control *P*. *aegyptiaca* through suicidal germination.

## 1. Introduction

Approximately 3500 to 4000 species of angiosperms lost their autotrophic lifestyle during evolution. These plants, which include broomrape (*Orobanche* and *Phelipanche* spp.), witchweed (*Striga spp*.), and dodder (*Cuscuta* spp.), now directly invade and parasitize other plants[1]. Severe infestations by these species can cause complete yield loss[2]. The Parasitic Plant Genome Project has sequenced the transcripts of three root-parasitic species (*P*. *aegyptiaca*, *S*. *hermonthica* and *Triphysaria versicolor*) at key life stages from seed conditioning through anthesis. The information gained from this project offers greater potential for increasing understanding not only about population dynamics, parasite virulence, and host resistance mechanisms but also about target sites for herbicide action and other novel control strategies[2].

Broomrapes are holoparasites, devoid of chlorophyll, and found largely in the Mediterranean and warm-temperate areas of Europe, North Africa, the Middle East, and northern China[3]. Broomrape species spend most of their life cycle underground. After germination, they form a haustorium which attaches to the roots and vascular tissues of host plants, facilitating the exchange of water, nutrients, hormones, toxins, and almost anything else able to travel through vascular connections[4]. Broomrape species affect legumes and to a lesser extent, a range of crops in Apiaceae, Asteraceae, Cucurbitaceae and Solanaceae. Crop losses range between 20 and 70% in many countries and regions[3]. Numerous physical, chemical, and biological approaches have been explored against broomrape, including soil solarization, organic amendment, and trap crops[5–8]. However, these control methods are generally ineffective and uneconomical [9,10]. Broomrape releases numerous small and long-living seeds. Reports indicate that topsoil may contain up to 4 million broomrape seeds m^−2^[4]. The seeds remain viable for up to 20 years in soil in the absence of a suitable host[4]. Broomrape seeds require chemical stimulants secreted by host roots to germinate, and the seedlings must contact the host root within a few days to survive [11]. It has been suggested that synthetic or natural chemicals could be used to reduce seedbanks by stimulating seed germination in the absence of suitable hosts. This control method (i.e., suicidal germination) is an attractive means of keeping seedbanks below a certain threshold. Many research projects are currently trying to identify natural or synthetic chemicals that can induce suicidal germination of broomrape.

Several types of chemical compounds [e.g., dihydrosorgoleone, glucosinolate derivatives, and strigolactones (SLs)], have been identified as chemical signals or germination stimulants for *Striga* and *Orobanche* [12,13]. Among these germination stimulants, SLs at concentrations of 10^−7^ to 10^−15^ mol/L result in the highest germination rates [1,5]. Treatment with a synthetic analogue of SL, GR24 (10^−7^ mol/L), resulted in *P*. *aegyptiaca* germination rates > 80%. However, *P*. *aegyptiaca* seeds must be conditioned for 4 to 7 d in a humid environment before GR24 can stimulate high germination rates. During conditioning, gibberellin (GA) is synthesized and plays an important role in subsequent germination[11]. Fluridone (FL), a carotenoid biosynthesis inhibitor, shortens the conditioning period required for *O*. *minor* seeds to germinate after stimulation by strigol. Fluridone also has the capacity to inhibit SL production and exudation in crops[14,15]. Gibberellic acid (GA_3_) and brassinolide influence the seed conditioning and germination of *Orobanche* and *Phelipanche* spp.[16]. A triazole-type SL-biosynthesis inhibitor, TIS108, can reduce the level of 2′-epi-5-deoxystrigol in rice. It has been hypothesized that TIS108 could potentially be applied to reduce the germination of root parasitic weeds [17].

In a preliminary experiment, it was observed that TIS108 and FL + GA_3_ both stimulated the rapid and high germination of *P*. *aegyptiaca* seeds without a water preconditioning period (i.e. hereafter referred to as unconditioned seeds). In contrast, GR24 required a water preconditioning period to stimulate *P*. *aegyptiaca* germination. The objective of this study was to better understand the seed germination mechanisms by comparing the transcriptome profiles of *P*. *aegyptiaca* seeds treated with TIS108-, FL+GA_3_-, and GR24.

## 2. Materials and methods

### 2.1. Plant material

Mature seeds of *P*. *aegyptiaca* were collected from a processing tomato field in 2016 at Junhu, Xinjiang Uyghur Autonomous Region, China. These seeds were stored at 4°C.

### 2.2. Seed germination tests

An improved culture method was used to compare the effects of various stimulants on the germination of unconditioned *P*. *aegyptiaca* seeds [14,18]. The *P*. *aegyptiaca* seeds were disinfected with 75% ethanol for 2 min and 1% sodium hypochlorite for 20 min [19]. The seeds were then rinsed five times with sterile water. Petri dishes were lined with two layers of filter paper and then autoclaved. Three glass fiber discs were laid on top of the filter paper. The sterilized unconditioned seeds were then spread evenly on the discs. A 100 μL aliquot of 1 of 14 treatment solutions was then applied to each disc in the petri dish. The treatment solutions included four combinations of FL + GA_3_ (100 mg/L FL + 1000, 100, 10, or 1 mg/L GA_3_). The 10 additional treatments included sterile water (negative control), GA_3_ (10 mg/L), abscisic acid (ABA, 1 mg/L), acetone (4 g/L), FL (100 mg/L), TIS108 (1000, 100, 10, and 1 mg/L) and GR24 (0.03 mg/L). The dishes were incubated in the dark at 25°C for seven days. In one additional treatment (positive control), conditioned seeds (sterile water for 7 d) were treated with 0.03 mg/L GR24 and then incubated for 5 d. All the seeds were then examined with a microscope to determine germination rates.

### 2.3. Transcriptome analysis

#### 2.3.1. Sample preparation

*P*. *aegyptiaca* seeds were surface sterilized and then spread evenly on a double layer of autoclaved filter paper in petri dishes (15 cm diam). The seeds in some dishes received no other treatment (unconditioned treatment). The remaining dishes were incubated in the dark at 25°C after treatment with 100 mg/L TIS108 (4 d incubation), 100 mg/L FL + 100 mg/L GA_3_ (4 d incubation), sterile water (7 d incubation), or sterile water (7 d incubation) followed by 0.03 mg/L GR24 (2 d incubation). These treatments will be respectively referred to as the TIS108, FL+GA_3_, conditioned, and GR24 treatments in the remainder of the paper. The unconditioned sample treated with water was the negative control and the conditioned sample treated with GR24 was the positive control. After incubation, the seeds were transferred from the glass fiber disks onto a piece of aluminum foil on a clean lab bench. The seeds were wrapped inside the foil, frozen in liquid N, and then stored at -80°C until RNA extraction.

#### 2.3.2. RNA extraction, RNA-seq library preparation, and sequencing

The total RNA of each sample was isolated using Trizol Reagent (Invitrogen, Gaithersburg, MD, USA) according to the manufacturer’s recommendations. High-quality RNA, with a 28S:18S ratio of more than 1.5 and a 260/280 absorbance ratio between 1.8 and 2.2, was used for library construction and sequencing. 3 μg RNA of each sample was used for the RNA sample preparations. Sequencing libraries were obtained using a NEBNext®Ultra™ RNA Library Prep Kit for Illumina® (NEB, USA) according to the manufacturer’s recommendations. The index codes were added to attribute sequences to each sample. In short, mRNA was purified from the total RNA by using poly-T oligo-attached magnetic beads. Fragmentation was carried out using divalent cations under elevated temperature in NEBNext First Strand Synthesis Reaction Buffer (5X). First strand cDNA was synthesized by random hexamer primer and M-MuLV Reverse Transcriptase (RNase H-). Subsequently, Second strand cDNA synthesis was performed using DNA Polymerase I and RNase H. Remaining overhangs were converted into blunt ends by exonuclease/polymerase activities. After adenylation of the DNA fragments 3′ ends, NEBNext Adaptors with hairpin loop structures were ligated to prepare for hybridization. In order to select cDNA fragments of 150~200 bp length, the library fragments were purified using AMPure XP system (Beckman Coulter, Beverly, USA). 3 μL of USER Enzyme (NEB, USA) was then used with size-selected, adaptor-ligated cDNA at 37°C for 15 min followed by 5 min at 95°C before PCR. PCR was then performed using Phusion High-Fidelity DNA polymerase, universal PCR primers and Index (X) Primer. Finally, the PCR products were purified (AMPure XP system) and the library quality was evaluated on the Agilent Bioanalyzer 2100 system. The Illumina sequencing platform was Hiseq X ten. The RNA library construction and sequencing were performed at Biomarker Technologies Co. Ltd., Beijing, China.

#### 2.3.3. Preprocessing of illumina reads and *de novo* transcriptome assembly

Raw data (raw reads) of fastq format were first processed through in-house perl scripts. In this step, clean data (clean reads) were obtained by removing reads containing adapters, reads containing poly-N, and low quality reads (i.e., reads where the Q-scores were <20 and the ratio of bases was >20%) from the raw data. The Q20, Q30, GC-content, and sequence duplication level of clean data were calculated simultaneously. High-quality clean data were used for downstream analyses. Transcriptome assembly was accomplished by Trinity [20].

#### 2.3.4. Functional annotation and differential expression analysis

Gene function was annotated using the following databases: non-redundant (NR) (National Center for Biotechnology Information NR protein sequences); Protein family (Pfam); EuKaryotic Orthologous Groups (KOG)/Clusters of Orthologous Groups of proteins (COG)/evolutionary genealogy of genes: Non-supervised Orthologous Groups (eggNOG); Swiss-Prot (A manually annotated and reviewed protein sequence database); Kyoto Encyclopedia of Genes and Genomes (KEGG); and Gene Ontology (GO). Bowtie was used to compare the sequenced reads with the unigene library. Gene expression levels were estimated by RSEM [21]. The clean data were mapped back to the assembled transcriptome and the read count for each gene was obtained from the mapping results. The FPKM value was used to express the corresponding unigene abundance. DESeq was used to identify the differentially expressed genes. The resulting *P* values were adjusted using Benjamini and Hochberg’s approach for controlling false discovery rate. Genes with an adjusted *P* value < 0.05 were assigned as differentially expressed. The False Discovery Rate (FDR) was used to determine the threshold of the *P* value in multiple tests. The cutoff thresholds for significance of expression were FDR < 0.01 and fold change ≥ 1.

#### 2.3.5. Endogenous hormone level analysis and validation of related genes

Endogenous hormone levels were determined using the frozen seed samples from each treatment in Section 2.3.1. The seed samples (1 g) were separately ground in liquid N using a mortar and pestle. The concentrations of ABA and GA were quantified using appropriate enzyme-linked immunosorbent assay kits (Chengling, Beijing, China) according to manufacturer’s instructions [11]. The ABA and GA concentrations are reported as the mean of three biological replicates. The RNA samples for RNA-seq were also used for real-time qRT-PCR validation. First-strand cDNA was synthesized using a Takara PrimeScript^TM^ RT Reagent Kit with gDNA Eraser (Perfect Real Time) (Dalian, China). Six plant-hormone-related genes, two SL perception-related genes, and two genes related to P450s were selected for validation of RNA-seq by qRT-PCR. *PaTubulin1* was used as an internal control [11,22]. The qRT-PCR was conducted using SYBR GreenER™ qPCR SuperMix Universal (Invitrogen, Gaithersburg, MD, USA). Thermal cycle conditions for PCR were as follows: 94°C for 3 min followed by 40 cycles at 94°C for 15 s and 59°C for 30 s. The expression level of the genes was calculated as the means of three biological replicates. Specific primers were designed using Primer 5.0 and are listed in S1 Table. All data were analyzed using one-way ANOVA (IBM SPSS Statistics 19.0, Armonk, NY, USA).

### 2.4. Accession numbers

RNA-seq data were submitted to NCBI under BioProject accession number PRJNA388245. The quality filtered and trimmed short read data set was deposited in the NCBI Sequence Read Archive (SRA) under accession numbers: SRR5680424, SRR5680425, SRR5680426, SRR5680427, SRR5680428, SRR5680429, SRR5680430, SRR5680431, SRR5680432, SRR5680433, SRR5680434, SRR5680435, SRR5680436, SRR5680437 and SRR5680438. The assembled transcripts can be accessed from NCBI Transcriptome Shotgun Assembly Sequence (TSA) Database under the accession number GFQM00000000.

## 3. Results

### 3.1. Seed germination rates

**Fig 1:**
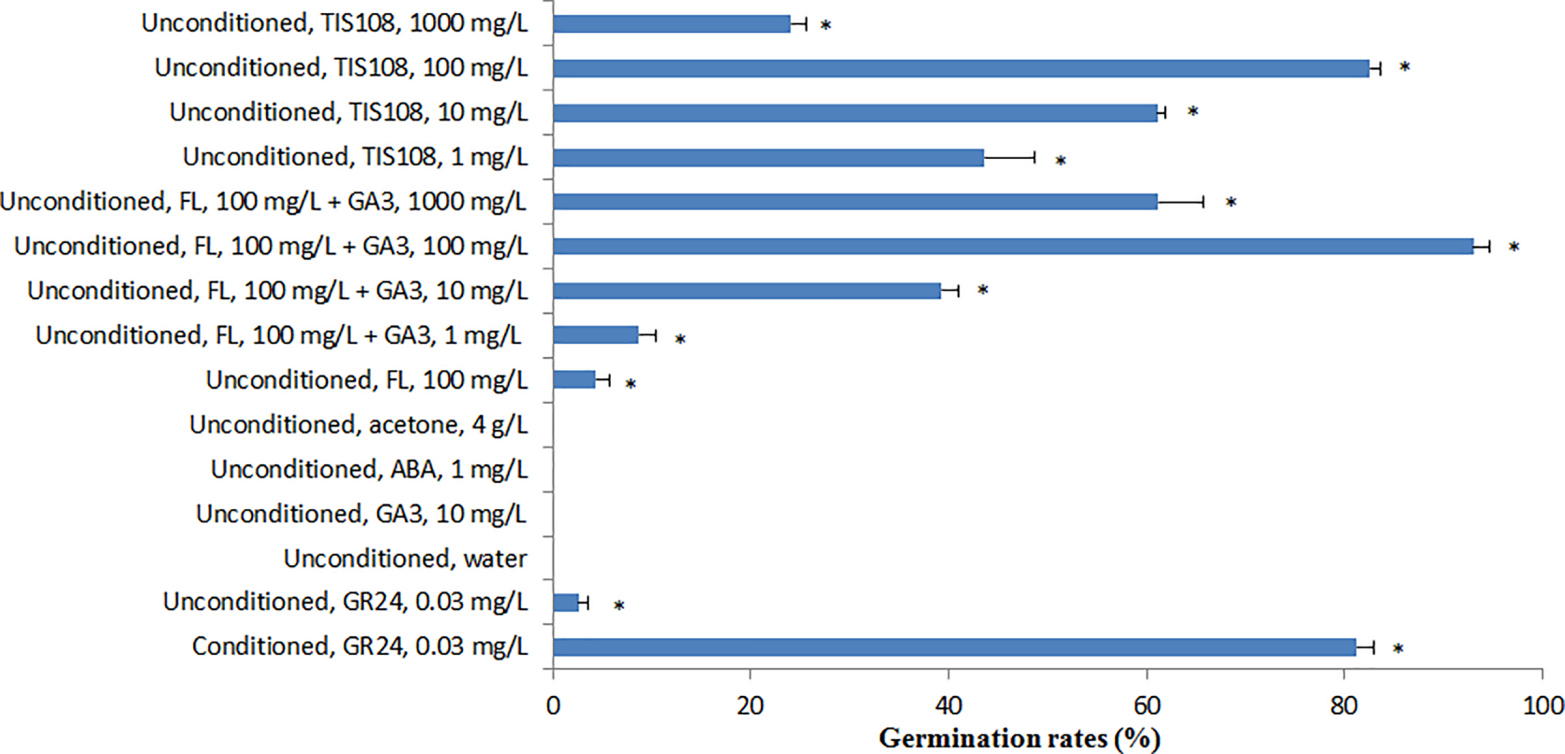
Germination rates of unconditioned *P*. *aegyptiaca* seeds as affected by water, gibberellic acid (GA_3_), GR24, acetone, fluridone (FL), and TIS108. Germination rates were determined after incubation in the dark at 25°C for 7 d. Conditioned seeds treated with GR24 and unconditioned seeds treated with water were used as positive and negative controls, respectively. Mean values ± standard deviations are from measurements on three independent germination assays. An asterisk (*) indicates that the values are significantly different than those in the water treatment according to Fisher’s protected LSD test (*P* <0.05).

**Fig 2:**
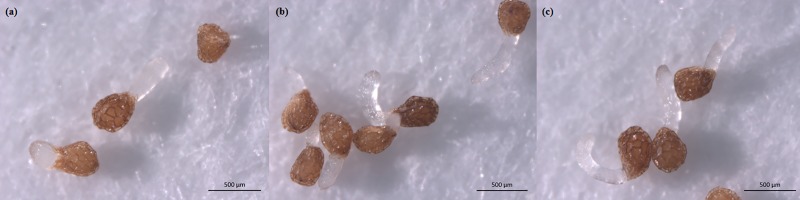
*P*. *aegyptiaca* germination. (a) Germinated and ungerminated seeds. Seeds were considered to have germinated when the radical length was more than half the seed diameter. (b) and (c) Germination of unconditioned *P*. *aegyptiaca* seeds after treatment with FL + GA_3_ and TIS108, respectively.

The germination rates of unconditioned *P*. *aegyptiaca* seeds were 0% when treated with water, GA_3_, ABA and acetone alone (Fig 1). The germination rate of unconditioned seeds treated with GR24 was 3%, whereas the germination rate of conditioned seeds treated with GR24 was 81%. Application of FL (100 mg/L) alone stimulated *P*. *aegyptiaca* germination; however the germination rate was only 4%. In comparison, FL + GA_3_ increased *P*. *aegyptiaca* germination significantly, with germination rates as high as 93% in the 100 mg/L FL+100 mg/L GA_3_ treatment. The SL biosynthesis inhibitor, TIS108, also promoted *P*. *aegyptiaca* germination, with germination rates reaching 83% in the 100 mg/L TIS108 treatment. More importantly, the FL + GA_3_ treatment and the TIS108 treatment both stimulated *P*. *aegyptiaca* germination without a water preconditioning period (Fig 2). It should be noted that there was acetone (as the solvent) in the solutions containing FL and TIS108. A preliminary experiment indicated that the acetone concentrations (0.04 to 40.00 g/L) in these solutions did not stimulate *P*. *aegyptiaca* germination. We chose to present the 4 g/L acetone treatment in Fig 1 because this was the acetone concentration in the treatments with the highest germination rates (FL 100 mg/L + GA3 100 mg/L and TIS108 100 mg/L).

### 3.2. RNA-seq and *de novo* assembly of the *P*. *aegyptiaca* transcriptome

**Fig 3:**
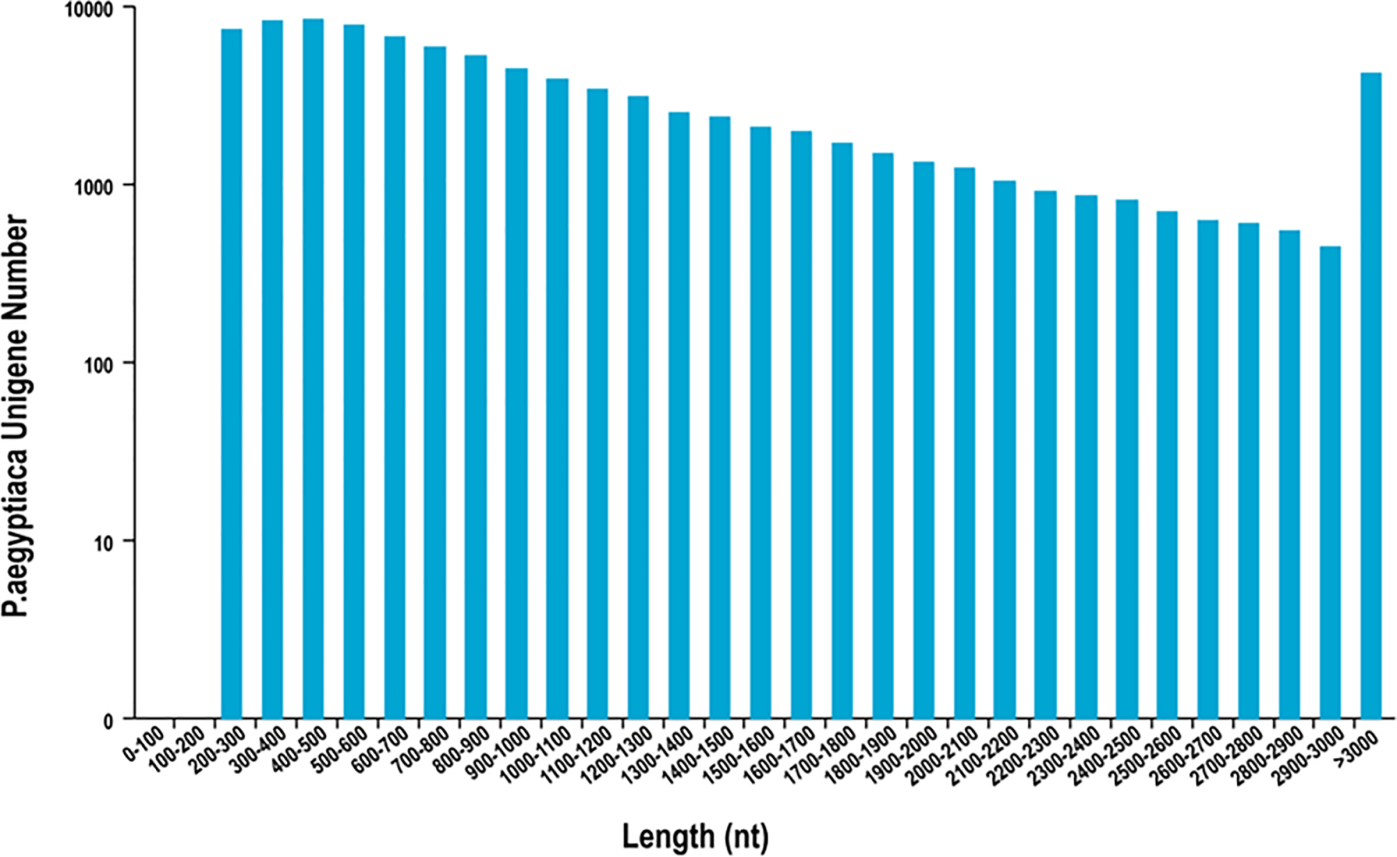
Unigene length distribution of *P*. *aegyptiaca* seeds.

To learn more about the germination mechanism, RNA-seq was performed using mRNA extracted from *P*. *aegyptiaca* seeds in five treatments: (i) unconditioned seeds, (ii) conditioned seeds, (iii) unconditioned seeds treated with TIS108, (iv) unconditioned seeds treated with FL+GA_3_, and (v) conditioned seeds treated with GR24. There were three biological replications of each treatment. After filtering, 429,090,579 pair-end reads of clean data were obtained. The percentage of Q30 bases in each sample was more than 94.90%, and the GC percentage ranged from 45.76% to 48.91% (S2 Table). Clean reads were *de novo* assembled using the Trinity. By combining unigenes from all 15 samples, 89,434 unigenes were assembled. The average N50 length of the unigenes was 1105.26 bp. The major length of the unigenes ranged from 300 to 2000 bp (Fig 3 and S3 Table). The clean reads for each sample was mapped to the library of assembled transcripts and unigenes. Mapped reads were used for subsequent analysis (Table 1).

**Table 1:** RNA-seq data and assembly results of *P*. *aegyptiaca* seeds as affected by different treatments. The number after each treatment name represents the replication number.

Samples	Clean Reads	Mapped Reads	Mapped Ratio
Unconditioned-1	21,312,030	13,682,131	64.20%
Conditioned, DI-water-1	23,760,435	15,338,029	64.55%
Unconditioned, FL+GA_3_-1	22,111,556	14,592,535	66.00%
Unconditioned, TIS108-1	25,793,060	16,708,666	64.78%
Conditioned, GR24-1	24,005,284	15,977,457	66.56%
Unconditioned-2	25,209,866	16,019,275	63.54%
Conditioned, DI-water-2	24,515,750	15,991,440	65.23%
Unconditioned, FL+GA_3_-2	25,539,216	16,587,180	64.95%
Unconditioned, TIS108-2	24,391,846	15,675,998	64.27%
Conditioned, GR24-2	21,836,698	14,021,636	64.21%
Unconditioned-3	22,107,194	14,123,756	63.89%
Conditioned, DI-water-3	21,551,978	13,684,833	63.50%
Unconditioned, FL+GA_3_-3	22,089,391	14,149,684	64.06%
Unconditioned, TIS108-3	21,954,428	14,215,757	64.75%
Conditioned, GR24-3	21,646,354	13,696,284	63.27%

### 3.3. Functional annotation of *P*. *aegyptiaca* unigenes

**Fig 4:**
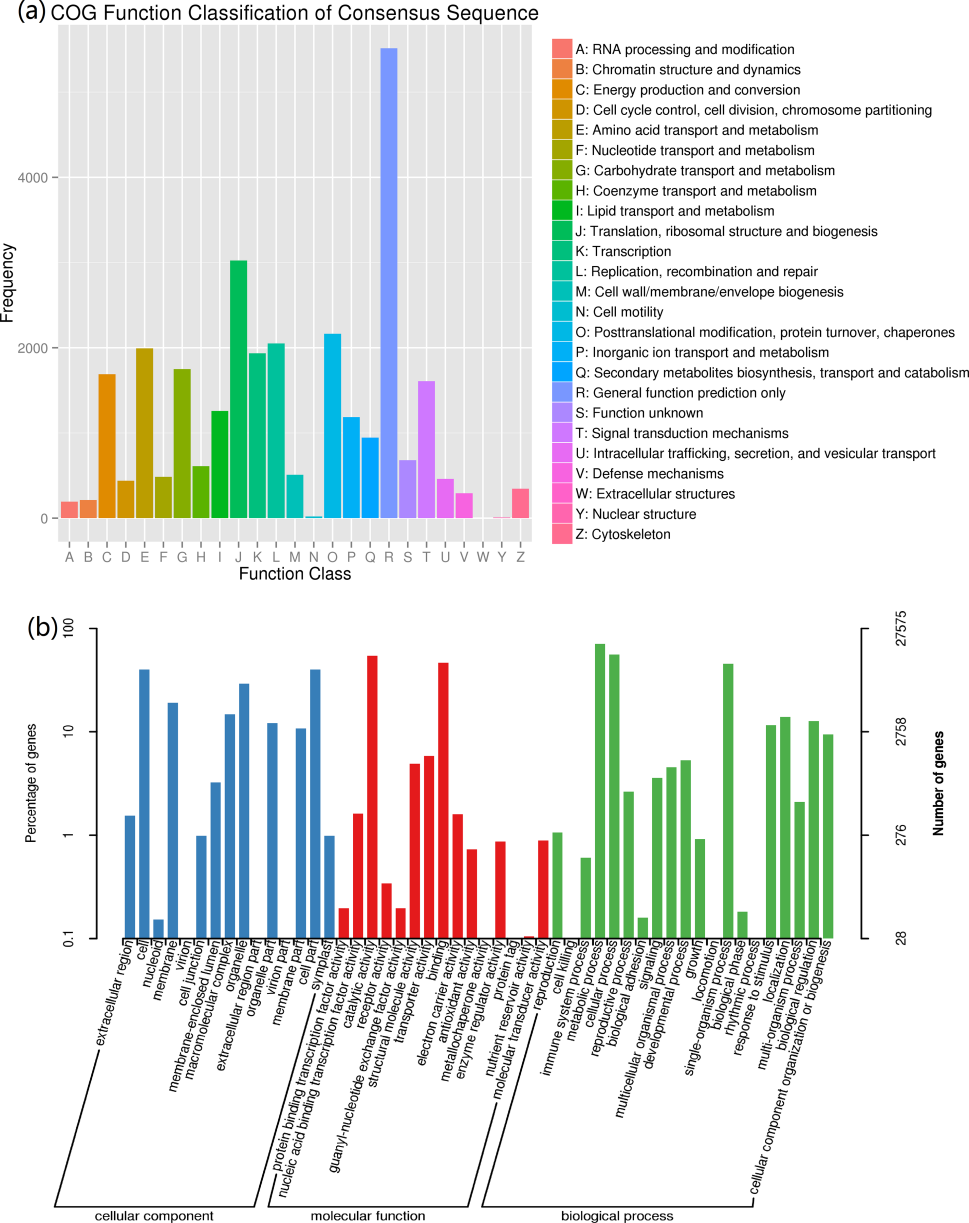
Functional classification of all unigenes in the COG and GO databases. (a) COG functional classification of the unigenes. (b) GO functional classification of the unigenes.

Functional annotation of *P*. *aegyptiaca* unigenes was conducted using BLAST software with an *E-*value ≤ 10^−5^. Among 89,434 non-redundant unigenes, there were 21,995 hits in COG, 27,575 hits in GO, 21,763 hits in KEGG, 33,118 hits in KOG, 27,364 hits in Swiss-Prot, 47,886 hits in eggNOG, and 52,917 hits in NR. The predicted amino acid sequences of the unigenes were subsequently compared with the Pfam database at an E-value ≤ 10^−10^. The HMMER software package was used to annotate the unigenes (S4 Table). The unigenes were classified into 25 groups based on COG function classification. The unigenes were mainly related to transcription and translation, including (i) translation, ribosomal structure, and biogenesis (3025); (ii) replication, recombination, and repair (2050); and (iii) post-translational modification, protein turnover, and chaperones (2164) (Fig 4A). Many unigenes were also related to transport and metabolism, especially the transport and metabolism of carbohydrates (1749), amino acids (1992), and lipids (1258). Some unigenes were involved in signal transduction mechanisms (1608), and cell cycle control, cell division, and chromosome partitioning (440) (Fig 4A).

According to GO function classification, the majority of unigenes under cellular component participate in cell (11,101), membrane (5302), macromolecular complex (5302), organelle (8042), organelle part (3348), membrane part (2961), and cell part (11,101). Molecular functions of these unigenes were mostly clustered in catalytic activity (15,042), structural molecule activity (1356), transporter activity (1607), and binding (12,843). The biological process categories were mainly metabolic process (19,482), cellular process (15,442), single-organism process (12,552), stimulus response (3222), localization (3845), and biological regulation (3507) (Fig 4B).

### 3.4. Comparative analysis of differential expression

**Fig 5:**
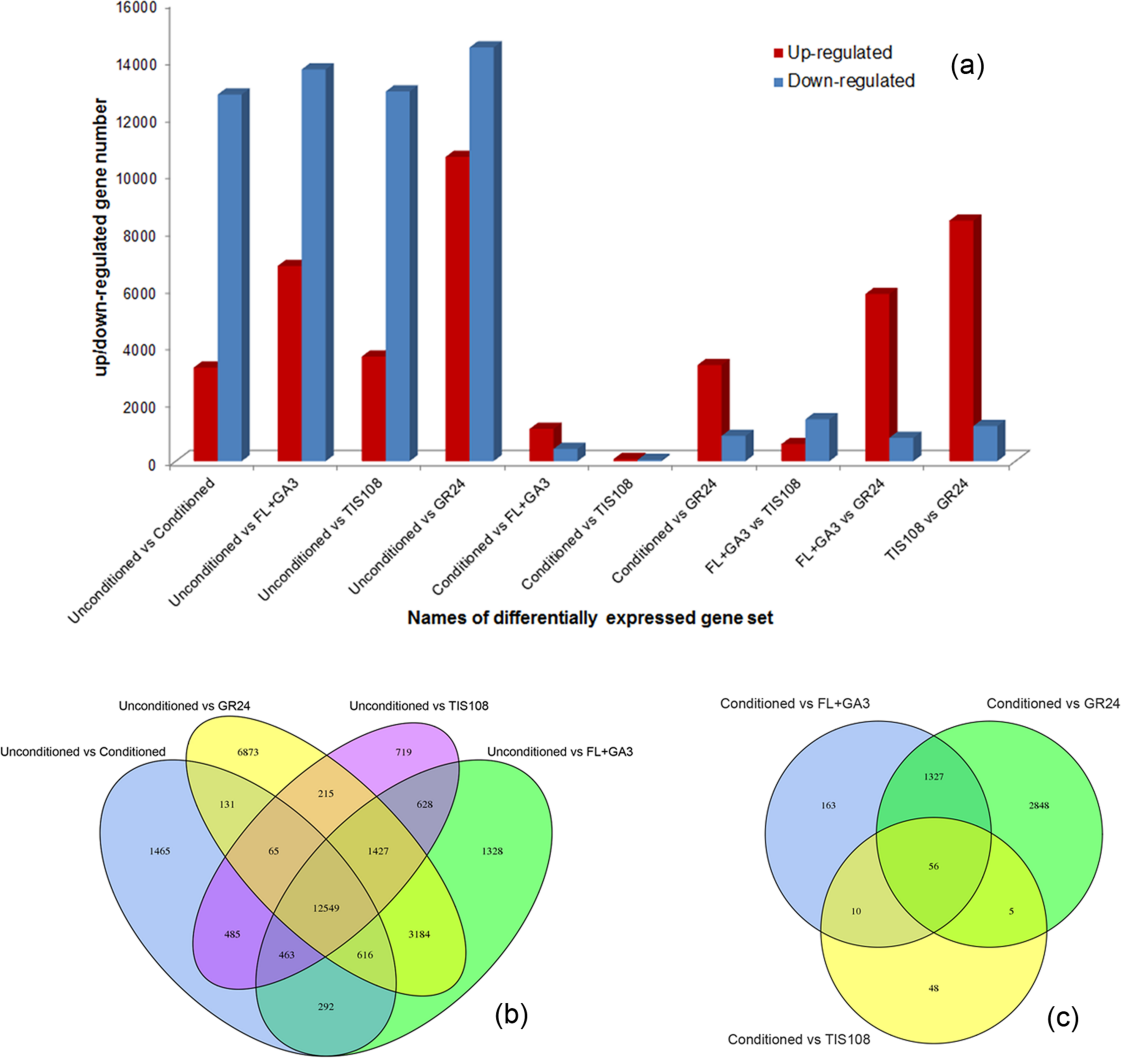
Statistical analysis of DEGs in *P*. *aegyptiaca* seeds as influenced by conditioning and germination stimulants. (a) Statistical analysis of up/down regulated unigenes in unconditioned, conditioned, FL+GA_3_-, TIS108-, and GR24 treated seeds. (b) Venn diagram of the DEGs in unconditioned vs conditioned, unconditioned vs GR24, unconditioned vs TIS108 and unconditioned vs FL+GA_3_. (c) Venn diagram of DEGs in conditioned vs GR24, conditioned vs TIS108, and conditioned vs FL+GA_3_.

Spatial analysis of differentially expressed unigenes (DEGs) was also performed to determine the degree of overlap between the five different treatments during seed germination. DESeq software was used to perform differential expression analysis between pairs of treatments to obtain a set of DEGs[23]. Compared with the unconditioned treatment, the number of up-regulated genes was 3268, 6814, 3650, and 10,620 in the conditioned, FL+GA_3_, TIS108, and GR24 treatments, respectively. The number of down-regulated genes was 12,798, 13,673, 12,901, and 14,440 in the conditioned, FL+GA_3_, TIS108, and GR24 treatments, respectively. Compared with conditioned samples, FL+GA_3_ contained 1,127 up-regulated and 429 down-regulated unigenes, TIS108 exhibited 74 up-regulated and 45 down-regulated unigenes, and GR24 showed 3356 up-regulated and 880 down-regulated unigenes. Compared with FL+GA_3_, TIS108 contained 596 up-regulated and 1464 down-regulated unigenes and GR24 had 5837 up-regulated and 818 down-regulated unigenes. Compared with TIS108, GR24 exhibited 8,394 up-regulated and 1,227 down-regulated unigenes (Fig 5A and S5 Table).

Different process genes were also compared using a Venn diagram. There were 16,066 DEGs in unconditioned vs unconditioned; 20,487 DEGs in unconditioned versus FL+GA_3_; 16,551 DEGs in unconditioned vs. TIS108; 25,060 DEGs in unconditioned vs GR24; 1,556 DEGs in conditioned vs FL+GA_3_; 119 DEGs in conditioned vs TIS108, and 4,236 DEGs in conditioned vs GR24 (Fig 5B and 5C). There were 12,549 DEGs among unconditioned vs conditioned, unconditioned vs FL+GA_3_, unconditioned vs TIS108, and unconditioned vs GR24 (Fig 5B). These results indicated that 12,549 genes were essential during germination of *P*. *aegyptiaca* seeds. There were 56 DEGs among conditioned versus FL+GA_3_, conditioned versus TIS108, and conditioned versus GR24 (Fig 5C). This suggested that these 56 genes may be key genes for *P*. *aegyptiaca* germination.

### 3.5. Pathways of DEGs based on KEGG database

KOBAS 2.0 software was used to test the significance (*P* <0.05) of the enrichment of DEGs in KEGG pathways[24]. The total number of DEGs annotated in the databases were as follows: 14,226 DEGs in unconditioned vs conditioned; 18,320 DEGs in unconditioned vs FL+GA_3_; 14,992 DEGs in unconditioned vs TIS108; 21,551 DEGs in unconditioned vs GR24; 1332 DEGs in conditioned vs FL+GA_3_; 79 DEGs in conditioned vs TIS108; 3476 DEGs in conditioned versus GR24; 1876 DEGs in FL+GA_3_ vs TIS108; 4887 DEGs FL+GA_3_ vs GR24; and 7503 DEGs in TIS108 vs GR24 (S6 Table). The KEGG annotation indicated that 128 pathways in unconditioned vs conditioned; 127 pathways in unconditioned vs FL+GA_3_, unconditioned vs TIS108, and unconditioned vs GR24; 99 pathways in conditioned versus FL+GA_3_, 20 pathways in conditioned vs TIS108, 123 pathways in conditioned versus GR24, 110 pathways in FL+GA_3_ vs TIS108, 121 pathways in FL+GA_3_ vs GR24, and 125 pathways in TIS108 vs GR24 were enriched. The pathways of unconditioned vs conditioned, unconditioned vs FL+GA_3_, unconditioned vs TIS108, and unconditioned vs GR24 were very similar. Only 20 pathways were significantly altered in conditioned vs TIS108 based on the KEGG database. These results indicated that majority of requirements for *P*. *aegyptiaca* germination were met after conditioning. Pathways related to metabolism (alpha-linolenic acid metabolism, pyrimidine metabolism, starch and sucrose metabolism, carbon metabolism, purine metabolism, and glutathione metabolism) and biosynthesis (aminoacyl-tRNA biosynthesis and sesquiterpenoid and triterpenoid biosynthesis) covered almost half of the DEGs. Many of the DEGs were involved in energy production processes such as oxidative phosphorylation, pentose phosphate pathway, and glycolysis/gluconeogenesis. Other processes included plant hormone signal transduction, plant–pathogen interaction, and ubiquitin-mediated proteolysis.

### 3.6. Role of GA in *P*. *aegyptiaca* germination

The KEGG annotation indicated six pathways related to GA biosynthesis in *P*. *aegyptiaca* seeds (S1A and S1B Fig). This suggested that GA plays an important role in *P*. *aegyptiaca* germination. Pairwise comparison (unconditioned vs conditioned, unconditioned vs FL+GA_3_, unconditioned vs TIS108, unconditioned vs GR24, conditioned vs FL+GA_3_, and conditioned vs GR24) resulted in the identification of five DEGs related to GA biosynthesis in *P*. *aegyptiaca*. The expressions of all five DEGs were significantly greater in the conditioned, GR24, TIS108, and FL+GA_3_ treatments than in the unconditioned treatment (Table 2). These results indicated that the five DEGs play important roles in the conditioning stage and germination of *P*. *aegyptiaca*.

**Table 2:** Key DEGs related to the endogenous hormones under unconditioned, conditioned, GR24, TIS108 and FL+GA_3_ treatments.

Gene ID	Gene name	Gene expressions (FPKM)					Annotation in KEGG
		Unconditioned	Conditioned	GR24	TIS108	FL+GA_3_	
c176949.graph_c0	*CPS*	0b	0.8283a	0.1006b	1.0013a	0.1211b	GA biosynthesis
c213846.graph_c0	*KS*	2.1824c	8.0045a	3.4058c	7.0654ab	6.1675b	GA biosynthesis
c212148.graph_c0	*KO*	20.5620d	39.1807c	60.6104a	40.1073bc	48.4856b	GA biosynthesis
c199689.graph_c0	*Ga20ox1-D*	0.6047e	17.3994a	7.6034c	13.5352b	5.1710d	GA biosynthesis
c190116.graph_c0	*GA3ox2*	0.0211d	0.3660d	5.1775a	1.6195c	3.1649b	GA biosynthesis
c206041.graph_c0	*PSY*	3.9156a	0.5036b	0.0397b	0.1961b	0.1250b	Carotene biosynthesis
c208625.graph_c0	*PDS*	2.1621a	0.2367b	0.1057b	0.3714b	0.4898b	Carotene biosynthesis
c157830.graph_c0	*crtISO*	4.4044e	9.7842d	13.4980b	11.0922c	15.8902a	Carotene biosynthesis
c206406.graph_c0	*D27*	1.2269c	3.8825a	2.6401b	2.4140b	2.6363b	SL biosynthesis
c214275.graph_c0	*NCED3*	11.5488b	12.3965b	3.7011c	28.3315a	11.5441b	ABA biosynthesis
c186185.graph_c0	*NCED2*	67.4027a	3.0454bc	1.2129c	9.9701b	3.0231bc	ABA biosynthesis
c196600.graph_c0	*CYP707A1*	0.1752d	1.9444bc	4.0840a	3.3379ab	1.3514cd	ABA catabolism
c220152.graph_c0	*ACO*	41.1838b	2.4058c	62.4597a	2.7025c	32.0591b	Ethylene biosynthesis
c178284.graph_c0	*ACO1*	9.3564a	0b	0b	0b	0.1150b	Ethylene biosynthesis
c172189.graph_c0	*ACO5-like*	0b	0.4073b	4.5449a	0.4551b	5.2078a	Ethylene biosynthesis
c217397.graph_c0	*CYP90B1*	0.494812b	1.169578b	4.223323a	1.191781b	3.699762a	Brassinosteroid biosynthesis
c203876.graph_c1	*CYP90C19*	0.179022d	1.496152d	17.70538a	3.866753c	14.95575b	Brassinosteroid biosynthesis
c91829.graph_c0	*CYP734A1*	0.305962b	1.361314b	6.643272a	2.313917b	6.136895a	Brassinosteroid biosynthesis
c208011.graph_c0	*CYP72A13*	10.00686a	0.785742c	4.046461b	0c	0.063386c	Brassinosteroid biosynthesis

*CPS*: ent-copalyl diphosphate synthase; *KS*: ent-kaurene synthase; *KO*: ent-kaurene oxidase; *Ga20ox1-D*: gibberellin 20-oxidase 1-D; *GA3ox2*: gibberellin biosynthesis-related protein GA3ox2; *PSY*: phytoene synthase; *PDS*: phytoene desaturase; *crtISO*: prolycopene isomerase; *D27*: beta-carotene isomerase D27; *NCED3*: 9-cis-epoxycarotenoid dioxygenase 3; *NCED2*: 9-cis-epoxycarotenoid dioxygenase 2; *CYP707A1*: ABA 8'-hydroxylase CYP707A1; *ACO*: ACC oxidase; *ACO1*: 1-aminocyclopropane-1-carboxylate oxidase 1; *ACO5-like*:1-aminocyclopropane-1-carboxylate oxidase 5-like; *CYP90B1*: cytochrome P450 90B1; *CYP90C19*: cytochrome P450 90C19; *CYP734A1*: cytochrome P450 734A1; *CYP72A13*: Cytochrome P450 72A13; Means within a line followed by the same letter does not differ significantly according to Fisher’s protected LSD test (*P*<0.05).

### 3.7. Role of ABA in *P*. *aegyptiaca* germination

The KEGG annotation indicated seven pathways related to ABA biosynthesis in *P*. *aegyptiaca* seeds (S1C Fig). This suggested that ABA, in addition to GA, also plays an important role in *P*. *aegyptiaca* germination. Pairwise comparison of the treatments (unconditioned vs conditioned, unconditioned vs FL+GA_3_, unconditioned vs TIS108, unconditioned vs GR24, FL+GA_3_ vs TIS108, FL+GA_3_ vs GR24, and TIS108 vs GR24) resulted in the identification of three DEGs related to GA biosynthesis in *P*. *aegyptiaca* (Table 2). This suggested that these three DEGs also participate in *P*. *aegyptiaca* germination. The expression of *CYP707A1* was significantly greater, but that of *NCED2* was significantly less, in the conditioned, GR24, TIS108, and FL+GA_3_ treatments compared with the unconditioned treatment. These results indicated that these genes are indispensable for *P*. *aegyptiaca* germination. The TIS108 treatment significantly increased *NCED3* expression compared with the unconditioned treatment, whereas the GR24 treatment had the opposite effect.

### 3.8. Other important genes related to germination

The KEGG pathway analysis revealed three DEGs (*PSY*, *PDS*, and *crtISO*) related to carotenoid biosynthesis, one DEG (*D27*) related to strigolactone biosynthesis, and three DEGs (*ACO*, *ACO1*, and *ACO5-like*) related to ethylene biosynthesis (Table 2 and S1D Fig). The expression of *PSY* and *PSD* was significantly less but the expression of *crtISO* and *D27* was significantly greater in the GR24, TIS108, and FL+GA_3_ treatments than in the unconditioned treatment (Table 2). The expression of *ACO* was significantly less in the TIS108, and FL+GA_3_ treatments but significantly greater in the GR24 treatment compared with the conditioned treatment. The expression of *ACO1* was significantly less, but the expression of *ACO5-like* was significantly greater in the conditioned, GR24, TIS108, and FL+GA_3_ treatments than in the unconditioned treatment. These results suggest that these genes are closely related to *P*. *aegyptiaca* germination. The KEGG pathway analysis also revealed some DEGs related to SL biosynthesis and signaling (Table 3) as well as to brassinosteroid (BR) biosynthesis (Table 2). When the expression of a gene was significantly less in the TIS108 treatment than in the other treatments, then such gene may be a potential target site of TIS108. Based on this theory, some potential target sites of TIS108 were obtained from the transcriptome data (Table 3).

**Table 3:** Expression of genes related to strigolactone and potential target site(s) of TIS108.

Gene ID	Gene name	Gene expressions (FPKM)					Annotation in KEGG
		Unconditioned	Conditioned	GR24	TIS108	FL+GA_3_	
c192595.graph_c0	*MAX2*	26.8149b	57.5734a	39.5196b	53.4968a	30.1277b	SL biosynthesis
c221871.graph_c0	*KAI2*	0.5237c	1.5407c	10.1703b	1.1781c	15.4844a	SL biosynthesis
c212844.graph_c1	*KAI2-like*	4.0092a	3.6226ab	1.3980d	2.7338bc	2.1810cd	SL biosynthesis
c211198.graph_c0	*D14-like*	2.6704c	7.4137bc	32.6106a	11.1315b	35.4002a	SL biosynthesis
c225931.graph_c0	*DAD2*	0.0824b	1.4485a	0.7500ab	0.8229ab	1.6309a	SL biosynthesis
c210504.graph_c0	*NADPH-cytochrome P450 reductase-like*	49.9144bc	54.1220b	73.8767a	41.7903c	57.7514b	potential target sites of TIS108
c202864.graph_c0	*Cytochrome P450 704C1-like*	30.5226a	29.7833a	28.7952a	16.8037b	20.1252b	potential target sites of TIS108
c185935.graph_c0	*Cytochrome P450 CYP736A12-like*	4.4690a	2.1528bc	3.2614ab	1.6387c	2.2211bc	potential target sites of TIS108
c224503.graph_c0	*Cytochrome P450 CYP81Q2*	2.2118a	0.0665bc	1.0619b	0c	0.0655bc	potential target sites of TIS108
c187679.graph_c0	*Cytochrome P450 71D13-like*	10.4351b	21.2041b	82.9333a	2.7094b	16.7248b	potential target sites of TIS108
c208011.graph_c0	*Cytochrome P450 72A13*	10.0069a	0.7857c	4.0465b	0c	0.0634c	potential target sites of TIS108
c209299.graph_c0	*Cytochrome P450 84A1-like *	3.2949b	3.7305b	20.0044a	1.6184b	19.2370a	potential target sites of TIS108
c145815.graph_c0	*Cytochrome P450 714C2-like*	0.4289b	0.4271b	2.9095a	0.1194b	0.3632b	potential target sites of TIS108
c219495.graph_c0	*Cytochrome P450 76A1-like*	86.7924a	64.9125ab	67.4746ab	17.5377c	39.6643bc	potential target sites of TIS108

Means within a line followed by the same letter do not differ significantly according to Fisher’s protected LSD test (*P*<0.05).

### 3.9. Endogenous hormone concentrations and validation of related genes by qRT-PCR

**Fig 6:**
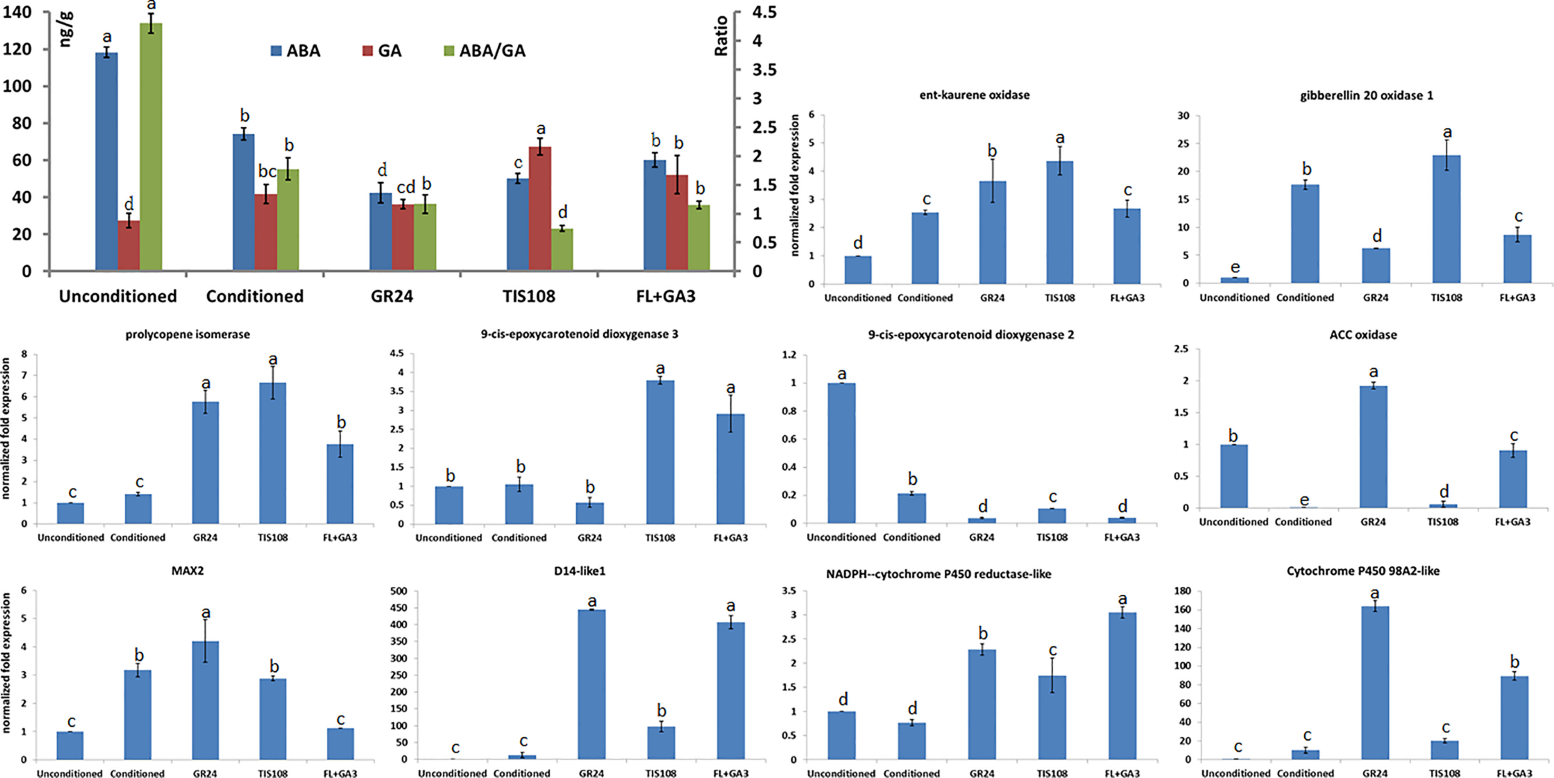
Endogenous ABA and GA concentrations and verification of the expression patterns of ten genes by qRT-PCR. The error bars indicate standard deviation. Different lowercase letters indicate significant differences among the treatments according to Fisher’s protected LSD test (P<0.05).

Endogenous ABA and GA concentrations were measured to learn more about the role of these hormones in *P*. *aegyptiaca* germination. The ABA concentrations were significantly less, but the GA was significantly greater in the conditioned, GR24, TIS108, and FL+GA_3_ treatments compared with the unconditioned treatment. The ABA:GA ratio steadily decreased during germination. This suggests that *P*. *aegyptiaca* can germinate only when the ABA:GA ratio declines to a certain value (Fig 6). The expression of ten representative genes was confirmed by qRT-PCR. The expression patterns of these genes were consistent with their transcriptional expression models (Fig 6).

## 4. Discussion

In this study, transcriptome sequencing was used to learn more about the germination mechanisms of *P*. *aegyptiaca* as affected by TIS108, FL+GA_3_, and GR24. By comparing treatments, several key genes and pathways were identified that are associated with *P*. *aegyptiaca* germination. Most of the DEGs were related to protein, DNA, RNA, energy biosynthesis, and metabolism. Only a few DEGs were involved in hormone biosynthesis; however, these DEGs are indispensable for seed germination.

### 4.1. Energy requirements for *P*. *aegyptiaca* germination

Physiological processes during germination require considerable energy. Lacking mineral absorption systems and photosynthetic apparatus, seeds depend on reserves such as starch, protein, and lipids to provide the necessary energy for germination[25–27]. Degradation products of energy reserves are constantly fed into glycolysis, followed by ATP synthesis through the TCA cycle, and mitochondrial electron transport [28]. In a previous study involving *P*. *aegyptiaca*, the adenylate energy charge (AEC) in GR24-treated seeds reached a maximum (0.9) after 1 d of conditioning and then remained constant for the next 9 d [29]. In the present study, two genes related to the glycolysis pathway (*PFK* and *PK*) were obtained in the transcriptome data of *P*. *aegyptiaca*. The expressions of *PFK* and *PK* were significantly down-regulated in the conditioned samples compared with unconditioned samples. These results indicated that most of the energy required for seed germination was generated during conditioning (S7 Table). During the early stage of seed germination, the energy for seed germination is mainly provided by glycolysis; however, during late germination, the energy provided by anaerobic respiration cannot meet the requirements of germinating seeds. At this point, the TCA cycle provides considerable energy under oxygen enrichment [30]. In the present study, the pyruvate dehydrogenase (*PDHA*) sequence, which is important in the TCA cycle, was obtained in the transriptome data. Compared with the unconditioned samples, *PDHA* expression was significantly up-regulated in the GR24, TIS108, and FL+GA_3_ treatments, suggesting that the energy required for later stages of germination is mainly provided by the TCA cycle.

### 4.2. Activation of endogenous hormone-related genes

Throughout the life cycle of plants, GAs regulate various developmental processes including seed development and seed germination[31]. The major bioactive GAs includes GA_1_, GA_3_, GA_4_, and GA_7_[32]. Previous studies showed that GA synthesis occurs during conditioning [33]. In this study, the following DEGs were annotated to the GA biosynthesis pathway: *CPS*, *KS*, *KO*, and *Ga20ox1-D*. Genes related to GA_1_, GA_3_, GA_4_, and GA_7_ syntheses were all up-regulated significantly in the conditioned, GR24, TIS108, and FL+GA_3_ treatments compared with the unconditioned treatment (Table 2). Previous studies showed that applying GA biosynthesis inhibitors during seed conditioning can suppress the germination of *Striga hermonthica*, *P*. *ramose*, and *P*. *aegyptiaca* in response to GR24[34–36]. Taken together, these results show that GA synthesis is an essential step during seed germination. This assumption is also supported by the increases in endogenous GA concentrations that were observed during *P*. *aegyptiaca* germination (Fig 5). The expression of *Ga20ox1-D* increased significantly in the order unconditioned < GR24 < conditioned (Table 2). This suggests that when GR24 is applied, the most important thing is not the synthesis of GA, but the degradation of ABA.

The hormones ABA and GA play antagonistic roles in regulating seed germination, and the concentrations of GA and ABA are negatively correlated in germinating seeds [37,38]. The GA is closely related to the promotion of seed germination. However, ABA inhibits this process. The antagonistic relationship and ratio of these two hormones regulate processes ranging from embryogenesis to seed germination [39]. Previous studies showed that exogenous ABA can inhibit *P*. *ramosa* germination [36]. The results of the present study also showed two significantly down-regulated DEGs associated with ABA biosynthesis and two up-regulated DEGs associated with ABA catabolism (Table 2). These results indicated that ABA is closely related to *P*. *aegyptiaca* germination. This idea was also supported by the decreases in endogenous ABA that were observed during *P*. *aegyptiaca* germination (Fig 5).

Previous studies showed that ethylene also plays an important role in seed germination, and this hormone neutralizes a number of negative functions of ABA during germination[40–44]. The interaction between GA and ethylene is complicated, as shown by their negative and positive reciprocal effects [45]. Three genes (*ACO*, *ACO1*, and *ACO5-like*) related to ethylene-directed synthesis precursors were observed in our data. However, the treatments in our study had inconsistent effects on these ethylene-related genes, increasing the expression of some of these genes and reducing the expression of others (Table 2). Therefore, further studies should be conducted to determine the role of ethylene in germination of *P*. *aegyptiaca* seeds.

### 4.3. Comparison of the three hormones involved in *P*. *aegyptiaca* germination

Previous studies showed that *P*. *aegyptiaca* seeds need to be conditioned at 20 to 26°C for several days in the dark before germination[11,46]. Researchers have proposed the following functions of the conditioning stage: (i) formation or activation of receptors for germination stimulants, (ii) leaching of germination inhibitors, and (iii) biosynthesis of plant hormones which may play important roles in germination [14,33,47]. Only 119 DEGs and 20 pathways were obtained in the transcriptome data of conditioned vs TIS08 (Fig 4). Seed germination rate tests showed that the TIS108 treatment but not the conditioned treatment can stimulate *P*. *aegyptiaca* germination (Fig 1). We suggest that the conditioned stage is important for seed germination, providing most, but not all of the conditions required for *P*. *aegyptiaca* germination.

Fluridone [i.e., 1-methyl-3-phenyl-5-[3-trifluoromethy1-(phenyl)]-4-(l*H*)-pyridinone] is an inhibitor of phytoene desaturase, which converts phytoene to phytofluene in the carotenoid biosynthesis pathway [48,49]. Carotenoids correspond to major precursors of ABA in plants; therefore FL also blocks ABA biosynthesis [50]. The transcriptome data in this study also showed this relationship between carotene biosynthesis and ABA biosynthesis (Table 2). The application of FL alone stimulated *P*. *aegyptiaca* germination to a small degree (Fig 1), indicating that a reduction in ABA increased *P*. *aegyptiaca* germination. The germination rate of *P*. *aegyptiaca* was significantly increased by the combined application of FL and GA. This suggested that *P*. *aegyptiaca* germination was caused by increasing GA_3_ concentration and decreasing ABA concentration. This phenomenon was also supported by transcriptome data (Table 2).

The synthetic analog of SL, GR24, can stimulate *P*. *aegyptiaca* germination [11,51]. Studies have shown that GR24 promotes the degradation of ABA and reduces its concentration in *P*. *ramosa* [29,52]. Other studies indicate that ABA and ethylene play important roles in *P*. *aegyptiaca* germination. It is also known that GR24 application dramatically changes ABA concentrations and the expression of ethylene-associated genes[11]. Analysis of the transcriptome data in this study showed that the expression of genes related to ABA biosynthesis and ethylene biosynthesis varied significantly among the five treatments (Table 2). The ABA concentrations were significantly less in the conditioned and GR24 samples than in the unconditioned samples (Fig 5). The ABA catabolic gene *CYP707A1* is a key component in the germination of *P*. *ramosa* seed treated with GR24 [29]. In the present study, *PaCYP707A1* was also obtained from the transcriptome data. The *PaCYP707A1* expression was significantly up-regulated in the conditioned, TIS108, FL+GA_3_, and GR24 samples compared with the unconditioned samples (Table 2). This indicated the importance of *PaCYP707A1* activation during the conditioning stage.

Researchers previously reported that TIS108, a triazole-type SL-biosynthesis inhibitor, reduced SL concentrations in Arabidopsis and rice [17,53]. Those authors suggested that the germination of root parasitic weeds could be reduced by treating the host plants with TIS108. The results of this study, however, indicated that TIS108 stimulated *P*. *aegyptiaca* germination rates to as high as 82.60% (Fig 1). This suggested that TIS108 can be used to control *P*. *aegyptiaca* by inducing suicidal germination. Previous studies showed that the target site(s) of TIS108 may be P450 (*MAX1*) homologues, and that TIS108 may affect some P450s involved in either the GA or BR biosynthesis pathways[17,53]. Our analysis of the transcriptome data suggested another potential target site (*CYP72A13*) in the BR biosynthesis pathway (Table 2).

### 4.4. Transcriptome annotation of *P*. *aegyptiaca* seeds

Only 62.63% of the filtered unigenes were annotated in our transcriptome data. These annotation results were similar to many other *de novo*-assembled transcriptomes[11,54–56]. The results showed that 18.53% of the unigenes were annotated to *Sesamum indicum*, 5.01% to *Erythranthe guttata*, and 4.91% to *Rhizopus delemar* (S2 Fig). A total of 119 DEGs were obtained when we compared conditioned with TIS108 samples. Only 79 of these DEGs were annotated, suggesting that 40 genes involved in germination may not be annotated and were possibly missed. These missing genes should be further studied in the future.

## 5. Conclusions

Transcriptome approaches were used to study the effects of TIS108, FL+GA_3_, and GR24 on *P*. *aegyptiaca* germination. Many DEGs related to seed germination were obtained. Some key genes associated with germination were detected. These genes were annotated as “hormone-associated”. The expression patterns of some important genes and the roles of hormones during seed germination were further verified by qRT-PCR analysis and endogenous hormone content analysis. The results showed that all three compounds (i.e., TIS108, FL+GA_3_, and GR24) stimulated *P*. *aegyptiaca* germination without a water preconditioning period. The common effect of all three compounds was that they reduced ABA concentrations in the seeds and increased GA concentrations. However, the target sites for these compounds differ. The results of this study provide information that is helpful for identifying other compounds that can stimulate *P*. *aegyptiaca* germination. We suggest that the important characteristic of these compounds is that they ultimately inhibit ABA synthesis or promote ABA degradation. This study also suggests that TIS108 or FL+GA_3_ could be used as an economical and effective means of controlling *P*. *aegyptiaca* by promoting suicidal germination.

## Supporting information

S1 Table Primers used in qRT-PCR. (DOCX)

S2 Table Evaluation of sample sequencing data. (DOCX)

S3 Table Unigene lengths in the assembled data. (DOCX)

S4 Table Unigene annotation. (DOCX)

S5 Table Number of DEGs. (DOCX)

S6 Table DEGs annotated in different databases. (DOCX)

S7 Table Key DEGs related to energy reserves in the unconditioned, conditioned, GR24, TIS108, and fluridone treatments. (DOCX)

S1 Fig (a) Pathway of gibberellic acid biosynthesis in unconditioned vs TIS108. (b) Pathway of gibberellic acid biosynthesis in unconditioned vs GR24. (c) Pathway of abscisic acid biosynthesis in unconditioned vs conditioned. (d) Pathway of ethylene biosynthesis in unconditioned vs FL+GA_3_.
(DOCX)

S2 Fig Species distribution annotated in the NR database. (DOCX)

## References

[1] Xie X, Yoneyama K, Yoneyama K. The strigolactone story. Annu Rev Phytopathol. 2010; 48: 93–117. doi: 10.1146/annurev-phyto-073009-114453 20687831 10.1146/annurev-phyto-073009-114453

[2] Westwood JH, Depamphilis CW, Das M, Fernández-Aparicio M, Honaas LA, Timko MP, et al The Parasitic Plant Genome Project: New tools for understanding the biology of *Orobanche* and *Striga*. Weed Sci. 2012; 60:295–306.

[3] Parker C. Observations on the current status of *Orobanche* and *Striga* problems worldwide. Pest Manag Sci. 2009; 65:453–459. doi: 10.1002/ps.1713 19206075 10.1002/ps.1713

[4] Joel DM, Hershenhorn J, Eizenberg H, Aly R, Ejeta G, Rich PJ, et al Biology and management of weedy root parasites. Hortic Rev. 2007; 8:267–350.

[5] Bouwmeester HJ, Matusova R, Zhongkui S, Beale MH. Secondary metabolite signalling in host-parasitic plant interactions. Curr Opin Plant Biol. 2003; 6:358–364. 12873531 10.1016/s1369-5266(03)00065-7

[6] Hershenhorn J, Eizenberg H, Dor E, Kapulnik Y, Goldwasser Y. *Phelipanche aegyptiaca* management in tomato. Weed Res. 2009; 49 (s1):34–47.

[7] Mauro RP, Monaco AL, Lombardo S, Restuccia A, Mauromicale G. Eradication of *Orobanche*/*Phelipanche* spp. seedbank by soil solarization and organic supplementation. Sci Hortic. 2015; 193:62–68.

[8] Pérez-de-Luque A, Eizenberg H, Grenz JH, Sillero JC, Ávila C, Sauerborn J, et al Broomrape management in faba bean. Field Crops Res. 2010; 115:319–328.

[9] Joel DM. The long-term approach to parasitic weeds control: manipulation of specific developmental mechanisms of the parasite. Crop Prot. 2000; 19:753–758.

[10] Goldwasser Y, Kleifeld Y. Recent approaches to *Orobanche* management. Weed Biol Manag. 2004; 6:439–466.

[11] Yao Z, Tian F, Cao X, Xu Y, Chen M, Xiang B, et al Global transcriptomic analysis reveals the mechanism of *Phelipanche aegyptiaca* seed germination. Int J Mol Sci. 2016; 17:1139–1158. 10.3390/ijms17071139 PMC4964512 27428962

[12] Chang M, Netzly DH, Butler LG, Lynn DG. Chemical regulation of distance. Characterization of the first natural host germination stimulant for *Striga asiatica*. J Am Chem Soc. 1986; 108:7858–7860. doi: 10.1021/ja00284a074 22283312 10.1021/ja00284a074

[13] Auger B, Pouvreau JB, Pouponneau K, Yoneyama K, Montiel G, Bizec BL, et al Germination stimulants of *Phelipanche ramosa* in the rhizosphere of Brassica napus are derived from the glucosinolate pathway. Mol Plant Microbe Interact. 2012; 25:993–1004. doi: 10.1094/MPMI-01-12-0006-R 22414435 10.1094/MPMI-01-12-0006-R

[14] Chae SH, Yoneyama K, Takeuchi Y, Joel DM. Fluridone and norflurazon, carotenoid-biosynthesis inhibitors, promote seed conditioning and germination of the holoparasite *Orobanche minor*. Physiol Plant. 2004; 120:328–337 doi: 10.1111/j.0031-9317.2004.0243.x 15032868 10.1111/j.0031-9317.2004.0243.x

[15] Matusova R., Rani K, Verstappen FW, Franssen MC, Beale MH, Bouwmeester HJ. The strigolactone germination stimulants of the plant-parasitic *Striga* and *Orobanche* spp. are derived from the carotenoid pathway. Plant Physiol. 2005; 139: 920–934. doi: 10.1104/pp.105.061382 16183851 10.1104/pp.105.061382 PMC1256006

[16] Takeuchi Y, Omigawa Y, Ogasawara M, Yoneyama K, Konnai M, Worsham D. Effects of brassinosteroids on conditioning and germination of clover broomrape (*Orobanche minor*) seeds. Plant Growth Regul. 1995; 16:153–160.

[17] Ito S, Umehara M, Hanada A, Kitahata N, Hayase H, Yamaguchi S, et al Effects of triazole derivatives on strigolactone levels and growth retardation in rice. PLoS ONE. 2011; 6(7):e21723 doi: 10.1371/journal.pone.0021723 21760901 10.1371/journal.pone.0021723 PMC3132747

[18] Sugimoto Y, Ueyama T. Production of (+)-5-deoxystrigol by Lotus japonicus root culture. Phytochemistry. 2008; 69:212–217. doi: 10.1016/j.phytochem.2007.06.011 17655890 10.1016/j.phytochem.2007.06.011

[19] Mangnus EM, Stommen PLA, Zwanenburg B. A standardized bioassay for evaluation of potential germination stimulants for seeds of parasitic weeds. J Plant Growth Regul. 1992; 11:91–98.

[20] Grabherr MG, Haas BJ, Yassour M, Levin JZ, Thompson DA, Amit I, et al Full-length transcriptome assembly from RNA-Seq data without a reference genome. Nat Biotechnol. 2011; 29: 644–652. doi: 10.1038/nbt.1883 21572440 10.1038/nbt.1883 PMC3571712

[21] Li B, Dewey CN. RSEM: accurate transcript quantification from RNA Seq data with or without a reference genome. BMC Bioinformatics. 2011; 12: 323–339. doi: 10.1186/1471-2105-12-323 21816040 10.1186/1471-2105-12-323 PMC3163565

[22] González-Verdejo CI, Die JV, Nadal S, Jiménez-Marín A, Moreno MT, Román B. Selection of housekeeping genes for normalization by real-time RT-PCR: analysis of *Or-MYB1* gene expression in *Orobanche ramosa* development. Anal Biochem. 2008; 379:38–43. 10.1016/j.ab.2008.05.003 18503743

[23] Leng N, Dawson JA, Thomson JA, Ruotti V, Rissman AI, Smits BMG, et al EBSeq: An empirical bayes hierarchical model for inference in RNA-seq experiments. Bioinformatics. 2013; 29:1035–1043. doi: 10.1093/bioinformatics/btt087 23428641 10.1093/bioinformatics/btt087 PMC3624807

[24] Xie C, Mao X, Huang J, Ding Y, Wu J, Dong S, et al KOBAS 2.0: a web server for annotation and identification of enriched pathways and diseases. Nucleic Acids Res. 2011; 39: W316–W322. doi: 10.1093/nar/gkr483 21715386 10.1093/nar/gkr483 PMC3125809

[25] Bewley JD. Seed germination and dormancy. Plant Cell.1997; 9:1055–1066. doi: 10.1105/tpc.9.7.1055 12237375 10.1105/tpc.9.7.1055 PMC156979

[26] Sheoran IS, Olson DJ, Ross AR, Sawhney VK. Proteome analysis of embryo and endosperm from germinating tomato seeds. Proteomics. 2005; 5:3752–3764. doi: 10.1002/pmic.200401209 16097031 10.1002/pmic.200401209

[27] Pritchard SL, Charlton WL, Baker A, Graham IA. Germination and storage reserve mobilization are regulated independently in *Arabidopsis*. Plant J. 2002; 31:639–647. 12207653 10.1046/j.1365-313x.2002.01376.x

[28] Weitbrecht K, Müller K, Leubner-Metzger G. First off the mark: early seed germination. J Exp Bot. 2011; 62:3289–3309. doi: 10.1093/jxb/err030 21430292 10.1093/jxb/err030

[29] Lechat MM, Pouvreau JB, Péron T, Gauthier M, Montiel G, Véronési C, et al *PrCYP707A1*, an ABA catabolic gene, is a key component of *Phelipanche ramosa* seed germination in response to the strigolactone analogue GR24. J Exp Bot. 2012; 63: 5311–5322. doi: 10.1093/jxb/ers189 22859674 10.1093/jxb/ers189 PMC3431000

[30] Yang P, Li X, Wang X, Chen H, Chen F, Shen S. Proteomic analysis of rice (*Oryza sativa*) seeds during germination. Proteomics. 2007; 7:3358–3368. doi: 10.1002/pmic.200700207 17849412 10.1002/pmic.200700207

[31] Sun TP, Gubler F. Molecular mechanism of gibberellin signaling in plants. Annu Rev Plant Biol. 2004; 55:197–223. doi: 10.1146/annurev.arplant.55.031903.141753 15377219 10.1146/annurev.arplant.55.031903.141753

[32] Yamaguchi S. Gibberellin metabolism and its regulation. Annu Rev Plant Biol. 2008; 59: 225–251. doi: 10.1146/annurev.arplant.59.032607.092804 18173378 10.1146/annurev.arplant.59.032607.092804

[33] Joel DM, Back A, Kleifeld Y, Gepstein S. Seed conditioning and its role in Orobanche seed germination: Inhibition by paclobutrazol. In K Wegmann, LJ Musselman, eds, Progress in Orobanche Research, Proceeding of the International Workshop on Orobanche Research. Obermachtal, Germany. 1991. pp 147–156.

[34] Song WJ, Zhou WJ, Jin ZL, Cao DD, Joel DM, Takeuchi Y, et al Germination response of *Orobanche* seeds subjected to conditioning temperature, water potential and growth regulator treatments. Weed Res. 2005; 45: 467–476.

[35] Uematsu K, Nakajima M, Yamaguchi I, Yoneyama K, Fukui Y. Role of cAMP in gibberellin promotion of seed germination in *Orobanche minor* Smith. J Plant Growth Regul. 2007; 26: 245–254.

[36] Zehhar N, Ingouff M, Bouya D, Fer A. Possible involvement of gibberellins and ethylene in *Orobanche ramosa* germination. Weed Res. 2002; 42:464–469.

[37] Batge SL, Ross JJ, Reid JB. Abscisic acid levels in seeds of the gibberellin-deficient mutant *lh-2* of pea (*Pisum sativum*). Physiol Plant. 1999; 105: 485–490.

[38] White CN, Proebsting WM, Hedden P, Rivin CJ. Gibberellins and seed development in maize. I. Evidence that gibberellin/abscisic acid balance governs germination versus maturation pathways. Plant Physiol. 2000; 122: 1081–1088. 10759503 10.1104/pp.122.4.1081 PMC58942

[39] Razem FA, Baron K, Hill RD. Turning on gibberellin and abscisic acid signaling. Curr Opin Plant Biol. 2006; 9: 454–459. doi: 10.1016/j.pbi.2006.07.007 16870490 10.1016/j.pbi.2006.07.007

[40] Linkies A, Muller K, Morris K, Tureckova V, Wenk M, Cadman CSC, et al Ethylene interacts with abscisic acid to regulate endosperm rupture during germination: a comparative approach using *Lepidium sativum* and *Arabidopsis thaliana*. Plant Cell. 2009; 21:3803–3822. doi: 10.1105/tpc.109.070201 20023197 10.1105/tpc.109.070201 PMC2814513

[41] Graeber K, Linkies A, Müller K, Wunchova A, Rott A, Leubner-Metzger G. Cross-species approaches to seed dormancy and germination: conservation and biodiversity of ABA-regulated mechanisms and the brassicaceae *DOG1* genes. Plant Mol Biol. 2010; 73: 67–87. doi: 10.1007/s11103-009-9583-x 20013031 10.1007/s11103-009-9583-x

[42] Kucera B, Cohn MA, Leubner-Metzger G. Plant hormone interactions during seed dormancy release and germination. Seed Sci Res. 2005; 15:281–307.

[43] Matilla AJ, Matilla-Vázquez MA. Involvement of ethylene in seed physiology. Plant Sci. 2008; 175:87–97.

[44] Morris K, Linkies A, Müller K, Oracz K, Wang X, Lynn JR, et al Regulation of seed germination in the close Arabidopsis relative *Lepidium sativum*: a global tissue-specific transcript analysis. Plant Physiol. 2011; 155: 1851–1870. doi: 10.1104/pp.110.169706 21321254 10.1104/pp.110.169706 PMC3091087

[45] Weiss D, Ori N. Mechanisms of cross talk between gibberellin and other hormones. Plant Physiol. 2007; 144:1240–1246. doi: 10.1104/pp.107.100370 17616507 10.1104/pp.107.100370 PMC1914132

[46] Joel DM, Bar H, Mayer AM, Plakhine D, Ziadne H, Westwood JH, et al Seed ultrastructure and water absorption pathway of the root-parasitic plant *Phelipanche aegyptiaca* (Orobanchaceae). Ann Bot. 2012; 109: 181–195. doi: 10.1093/aob/mcr261 22025523 10.1093/aob/mcr261 PMC3241583

[47] Bar-Nun N, Mayer AM. Composition of and changes in storage compounds in *Orobanche aegyptiaca* seeds during preconditioning. Isr J Plant Sci. 2002; 50:277–279.

[48] Bartels PG, Watson CW. Inhibition of carotenoid synthesis by fluridone and norflurazon. Weed Sci. 1978; 26:198–203.

[49] Fong F, Schiff JA. Blue-light-induced absorbance changes associated with carotenoids in *Euglena*. Planta.1979; 146:119–127. doi: 10.1007/BF00388221 24318048 10.1007/BF00388221

[50] Quatrano RS, Bartels D, Ho TD, Pages M. New insights into ABA-mediated processes. Plant Cell. 1997; 9:470–475.

[51] Malik H, Rutjes FPJT, Zwanenburg B. A new efficient synthesis of GR24 and dimethyl A-ring analogues, germinating agents for seeds of the parasitic weeds Striga and *Orobanche* spp. Tetrahedron. 2010; 66:7198–7203.

[52] Lechat MM, Brun G, Montiel G, Véronési C, Simier P, Thoiron S, et al Seed response to strigolactone is controlled by abscisic acid-independent DNA methylation in the obligate root parasitic plant, *Phelipanche ramosa* L. Pomel. J Exp Bot. 2015; 66:3129–3140. doi: 10.1093/jxb/erv119 25821070 10.1093/jxb/erv119 PMC4449535

[53] Ito S, Umehara M, Hanada A, Yamaguchi S, Asami T. Effects of strigolactone-biosynthesis inhibitor TIS108 on *Arabidopsis*. Plant Signal Behav. 2013; 8(5):e24193 doi: 10.4161/psb.24193 23511201 10.4161/psb.24193 PMC3907539

[54] Liu S, Li W, Wu Y, Chen C, Lei J. *De novo* transcriptome assembly in Chili Pepper (*Capsicum frutescens*) to identify genes involved in the biosynthesis of capsaicinoids. PloS ONE. 2013, 8(1):e48156 doi: 10.1371/journal.pone.0048156 23349661 10.1371/journal.pone.0048156 PMC3551913

[55] Nakasugi K, Crowhurst RN, Bally J, Wood CC, Hellens RP, Waterhouse PM. *De novo* transcriptome sequence assembly and analysis of RNA silencing genes of *Nicotiana benthamiana*. PloS ONE. 2013; 8(3):e59534 doi: 10.1371/journal.pone.0059534 23555698 10.1371/journal.pone.0059534 PMC3610648

[56] Ranjan A, Ichihashi Y, Farhi M, Zumstein K, Townsley B, David-Schwartz R, et al *De novo* assembly and characterization of the transcriptome of the parasitic weed dodder identifies genes associated with plant parasitism. Plant Physiol. 2014; 166: 1186–1199. doi: 10.1104/pp.113.234864 24399359 10.1104/pp.113.234864 PMC4226353

