# Supplementary material for: Transcriptome analysis of Phelipanche aegyptiaca seed germination mechanisms stimulated by fluridone, TIS108, and GR24
Source: PLoS One. 2017 Nov 3;12(11):e0187539. doi: 10.1371/journal.pone.0187539 (PMC5669479; doi:10.1371/journal.pone.0187539)
Supplement: S1 Table — (DOCX) [file pone.0187539.s001.docx]

**S1 Table. Primers used in qRT-PCR**

| Gene | Primer | Forward and reverse primers 5’ – 3’ |
| --- | --- | --- |
| *Patublin1* | Sense | GGTCCCGAAAGATGTCAACGC |
|  | Anti-sense | GAGAACACCTCCGCCACGCT |
| *ent-kaurene oxidase* | Sense | GGTGCCATTGAGGTGGATTG |
|  | Anti-sense | CATCACAGACTGCCTACGGAAA |
| *gibberellin 20 oxidase 1* | Sense | GGACACTTGGCTGGGGACTA |
|  | Anti-sense | CGTGGATTTATGGAGGTTTTCA |
| *prolycopene isomerase* | Sense | CAGTGGTCGGGTCAGGTATG |
|  | Anti-sense | CTGGAGGAAGTTCTGGGTTTTA |
| *9-cis-epoxycarotenoid dioxygenase 3* | Sense | TGAGGGTGGGATTATGGGTC |
|  | Anti-sense | TTATGGAACGCTTGGGAGG |
| *9-cis-epoxycarotenoid dioxygenase 2* | Sense | GGCTGCTTTTGCTTCCATC |
|  | Anti-sense | ACCCTCGTCGCATTCGTTG |
| *ACC oxidase* | Sense | CGCTGACCTTATCGTCCTGG |
|  | Anti-sense | GCACCAAAGTGAGCAAATACCC |
| *MAX2* | Sense | GACCGAGACAGAAATCAAGGC |
|  | Anti-sense | CTGAATCTGTTGAACCCATCGT |
| *D14-like* | Sense | TGGGGCATTCATTGTCGG |
|  | Anti-sense | GCCACCGTTGTAACCCTTGTC |
| *NADPH--cytochrome P450 reductase-like* | Sense | AGAACTGAAGCCTTTTGTGCC |
|  | Anti-sense | CTTGGTCCTGCCGTATTATTTT |
| *Cytochrome P450 98A2-like* | Sense | AAGATTCCAAGGGTCCAAAAGA |
|  | Anti-sense | AGGGAGCATTAGAGGGGTCG |
