# Supplementary material for: Transcriptome analysis of Phelipanche aegyptiaca seed germination mechanisms stimulated by fluridone, TIS108, and GR24
Source: PLoS One. 2017 Nov 3;12(11):e0187539. doi: 10.1371/journal.pone.0187539 (PMC5669479; doi:10.1371/journal.pone.0187539)
Supplement: S2 Table — (DOCX) [file pone.0187539.s002.docx]

**S2 Table. Evaluation of sample sequencing data**

| Samples | Read Sum | Base Sum | GC(%) | N(%) | Q20(%) | Cycle Q20(%) | Q30(%) |
| --- | --- | --- | --- | --- | --- | --- | --- |
| Unconditioned-1 | 21,312,030 | 6,346,243,996 | 47.85 | 0 | 97.76 | 100 | 95.3 |
| Conditioned, DI-water-1 | 23,760,435 | 7,078,987,924 | 47.64 | 0 | 97.67 | 100 | 95.11 |
| Unconditioned, FL+GA_3_-1 | 27,644,844 | 8,238,902,948 | 46.90 | 0 | 97.91 | 100 | 95.53 |
| Unconditioned, TIS108-1 | 25,793,060 | 7,691,005,742 | 48.03 | 0 | 97.61 | 100 | 94.99 |
| Conditioned, GR24-1 | 30,009,972 | 8,957,623,360 | 47.12 | 0 | 97.94 | 100 | 95.63 |
| Unconditioned-2 | 25,209,866 | 7,510,866,714 | 48.09 | 0 | 97.56 | 100 | 94.94 |
| Conditioned, DI-water-2 | 24,515,750 | 7,311,816,898 | 46.61 | 0 | 97.77 | 100 | 95.31 |
| Unconditioned, FL+GA_3_-2 | 25,539,216 | 7,617,963,892 | 46.65 | 0 | 97.74 | 100 | 95.23 |
| Unconditioned, TIS108-2 | 24,391,846 | 7,273,223,704 | 47.05 | 0 | 97.64 | 100 | 95.1 |
| Conditioned, GR24-2 | 32,117,199 | 9,582,830,396 | 47.95 | 0 | 97.55 | 100 | 94.9 |
| Unconditioned-3 | 38,113,197 | 11,345,293,930 | 48.07 | 0 | 97.75 | 100 | 95.3 |
| Conditioned, DI-water-3 | 43,094,110 | 12,843,525,056 | 48.91 | 0 | 97.72 | 100 | 95.21 |
| Unconditioned, FL+GA_3_-3 | 29,456,154 | 8,782,740,996 | 45.76 | 0 | 97.88 | 100 | 95.5 |
| Unconditioned, TIS108-3 | 29,275,896 | 8,727,343,290 | 46.93 | 0 | 97.73 | 100 | 95.21 |
| Conditioned, GR24-3 | 28,857,004 | 8,612,260,424 | 48.25 | 0 | 97.79 | 100 | 95.32 |

Samples: The analysis number of sample; Read Number: Total number of pair-end reads of clean data; Base Number: Total base number of clean data; GC%: GC content of clean data; Q30%: Percentage of bases with mass values greater than or equal to 30 of clean data.
