# Supplementary material for: Transcriptome analysis of Phelipanche aegyptiaca seed germination mechanisms stimulated by fluridone, TIS108, and GR24
Source: PLoS One. 2017 Nov 3;12(11):e0187539. doi: 10.1371/journal.pone.0187539 (PMC5669479; doi:10.1371/journal.pone.0187539)
Supplement: S3 Table — (DOCX) [file pone.0187539.s003.docx]

**S3 Table. Unigene lengths in the assembled data**

| Length Range | Transcript | Unigene |
| --- | --- | --- |
| 200-300 | 10,649(6.25%) | 7,380(8.25%) |
| 300-500 | 26,613(15.61%) | 16,563(18.52%) |
| 500-1000 | 53,918(31.63%) | 29,948(33.49%) |
| 1000-2000 | 51,297(30.09%) | 23,676(26.47%) |
| 2000+ | 27,982(16.42%) | 11,867(13.27%) |
| Total Number | 170,459 | 89,434 |
| Total Length | 208,947,894 | 98,847,879 |
| N50 Length | 1,677 | 1,524 |
| Mean Length | 1225.80 | 1105.26 |

Length Range: the length of unigenes; Total Number: the total number of assembled unigenes; Total Length: the total length of the assembled unigenes; N50 Length: the length of unigene's N50; Mean Length: the average length of unigenes.
