# Supplementary material for: Transcriptome analysis of Phelipanche aegyptiaca seed germination mechanisms stimulated by fluridone, TIS108, and GR24
Source: PLoS One. 2017 Nov 3;12(11):e0187539. doi: 10.1371/journal.pone.0187539 (PMC5669479; doi:10.1371/journal.pone.0187539)
Supplement: S4 Table — (DOCX) [file pone.0187539.s004.docx]

**S4 Table. Unigene annotation**

| Annotated Database | Annotated Number | 300<=length<1000 | length>=1000 |
| --- | --- | --- | --- |
| COG_Annotation | 21,995 | 9,254 | 11,920 |
| GO_Annotation | 27,575 | 12,029 | 14,326 |
| KEGG_Annotation | 21,763 | 9,673 | 11,234 |
| KOG_Annotation | 33,118 | 13,632 | 18,312 |
| Pfam_Annotation | 41,500 | 16,723 | 23,651 |
| Swissprot_Annotation | 27,364 | 10,503 | 15,968 |
| eggNOG_Annotation | 47,886 | 20,688 | 25,326 |
| nr_Annotation | 52,917 | 22,861 | 27,952 |
| All_Annotated | 56,010 | 24,551 | 29,110 |
