# Supplementary material for: Transcriptome analysis of Phelipanche aegyptiaca seed germination mechanisms stimulated by fluridone, TIS108, and GR24
Source: PLoS One. 2017 Nov 3;12(11):e0187539. doi: 10.1371/journal.pone.0187539 (PMC5669479; doi:10.1371/journal.pone.0187539)
Supplement: S5 Table — (DOCX) [file pone.0187539.s005.docx]

**S5 Table. Number of DEGs**

| DEG Set | All DEG | Up-regulated | Down-regulated |
| --- | --- | --- | --- |
| Unconditioned vs Conditioned | 16,066 | 3,268 | 12,798 |
| Unconditioned vs FL+GA_3_ | 20,487 | 6,814 | 13,673 |
| Unconditioned vs TIS108 | 16,551 | 3,650 | 12,901 |
| Unconditioned vs GR24 | 25,060 | 10,620 | 14,440 |
| Conditioned vs FL+GA_3_ | 1,556 | 1,127 | 429 |
| Conditioned vs TIS108 | 119 | 74 | 45 |
| Conditioned vs GR24 | 4,236 | 3,356 | 880 |
| FL+GA_3_ vs TIS108 | 2,060 | 596 | 1,464 |
| FL+GA_3_vs GR24 | 6,655 | 5,837 | 818 |
| TIS108 vs GR24 | 9,621 | 8,394 | 1,227 |

DEG Set: the name of a differentially expressed gene set; All DEG: the number of DEGs; up-regulated: the number of up-regulated genes; down-regulated: the number of down-regulated genes.
