# Supplementary material for: Transcriptome analysis of Phelipanche aegyptiaca seed germination mechanisms stimulated by fluridone, TIS108, and GR24
Source: PLoS One. 2017 Nov 3;12(11):e0187539. doi: 10.1371/journal.pone.0187539 (PMC5669479; doi:10.1371/journal.pone.0187539)
Supplement: S6 Table — (DOCX) [file pone.0187539.s006.docx]

**S6 Table.** **DEGs annotated in different databases**

| DEG Set | Annotated | COG | GO | KEGG | KOG | Pfam | Swiss-Prot | eggNOG | nr |
| --- | --- | --- | --- | --- | --- | --- | --- | --- | --- |
| Unconditioned vs Conditioned | 14,226 | 6,422 | 8,679 | 5,634 | 8,814 | 11,257 | 7,253 | 12,790 | 14,141 |
| Unconditioned vs FL+GA_3_ | 18,320 | 8,268 | 10,804 | 7,452 | 11,403 | 14,803 | 9,886 | 16,427 | 18,037 |
| Unconditioned vs TIS108 | 14,992 | 7,072 | 9,121 | 6,184 | 9,514 | 12,174 | 7,683 | 13,507 | 14,800 |
| Unconditioned vs GR24 | 21,551 | 9,816 | 12,023 | 9,061 | 13,645 | 17,724 | 11,595 | 19,277 | 20,873 |
| Conditioned vs FL+GA_3_ | 1,332 | 526 | 783 | 552 | 730 | 1,107 | 1,000 | 1,182 | 1,305 |
| Conditioned vs TIS108 | 79 | 24 | 36 | 23 | 39 | 60 | 48 | 62 | 75 |
| Conditioned vs GR24 | 3,476 | 1,315 | 1,869 | 1,423 | 1,980 | 2,849 | 2,396 | 3,050 | 3,347 |
| FL+GA_3_ vs TIS108 | 1,876 | 712 | 1,104 | 727 | 1,035 | 1,548 | 1,416 | 1,668 | 1,858 |
| FL+GA_3_ vs GR24 | 4,887 | 2,201 | 1,861 | 2,224 | 3,227 | 4,336 | 2,687 | 4,235 | 4,355 |
| TIS108 vs GR24 | 7,503 | 3,172 | 3,487 | 3,355 | 4,776 | 6,405 | 4,755 | 6,572 | 6,995 |

DEG Set: the name of a differentially expressed gene set; Annotated: the number of DEGs annotated in all databases; The third column to the last column indicates the number of DEGs annotated in the database.
