# Supplementary material for: Transcriptome analysis of Phelipanche aegyptiaca seed germination mechanisms stimulated by fluridone, TIS108, and GR24
Source: PLoS One. 2017 Nov 3;12(11):e0187539. doi: 10.1371/journal.pone.0187539 (PMC5669479; doi:10.1371/journal.pone.0187539)
Supplement: S7 Table — (DOCX) [file pone.0187539.s007.docx]

S7 Table. **Key DEGs related to energy reserves in the unconditioned, conditioned, GR24, TIS108, and fluridone treatments**

| Gene ID | Gene name | Gene expressions | | | | | Annotation in KEGG |
| --- | --- | --- | --- | --- | --- | --- | --- |
|  |  | Dormant | Conditioned | GR24 | TIS108 | FL+GA_3_ |  |
| c185111.graph_c0 | *PFK5* | 10.8555 | 8.6179 | 53.918 | 10.5320 | 35.1231 | Glycolysis pathway |
| c139113.graph_c1 | *PFK2* | 164.4177 | 77.2356 | 16.7779 | 84.5120 | 22.2108 | Glycolysis pathway |
| c207292.graph_c0 | *PK* | 20.47866 | 2.94289 | 0.54271 | 2.21270 | 4.39025 | Glycolysis pathway |
| c207123.graph_c0 | *PK* | 79.35756 | 59.0238 | 196.1262 | 62.0075 | 160.1462 | Glycolysis pathway |
| c161849.graph_c0 | *PK isozyme A* | 36.6088 | 38.6619 | 86.2317 | 40.7856 | 82.5975 | Glycolysis pathway |
| c140016.graph_c0 | *PDHA E1 component alpha subunit* | 0.0181 | 5.6192 | 10.9220 | 0.5035 | 0.8770 | Tricarboxylic Acid Cycle |
| c205744.graph_c0 | *PDHA E1 component subunit alpha* | 0.0591 | 7.9672 | 6.8373 | 21.7466 | 11.1224 | Tricarboxylic Acid Cycle |
| c126894.graph_c1 | *PDHA E1 component subunit beta* | 0 | 4.6403 | 8.1082 | 0.5927 | 0.4264 | Tricarboxylic Acid Cycle |
| c212238.graph_c1 | *PDHA E1 component subunit beta-1* | 0.0320 | 13.1890 | 9.0699 | 22.8615 | 10.6196 | Tricarboxylic Acid Cycle |
| c219822.graph_c0 | *PDHA E1 component subunit beta-3* | 11.5117 | 5.8867 | 39.9622 | 6.4360 | 30.4308 | Tricarboxylic Acid Cycle |

*PFK*: 6-phosphofructokinase; *PK*: pyruvate kinase; *PDHA*: pyruvate dehydrogenase;
