# Supplementary material for: Transcriptome analysis of Phelipanche aegyptiaca seed germination mechanisms stimulated by fluridone, TIS108, and GR24
Source: PLoS One. 2017 Nov 3;12(11):e0187539. doi: 10.1371/journal.pone.0187539 (PMC5669479; doi:10.1371/journal.pone.0187539)

**S1 Fig. (a) Pathway of gibberellic acid biosynthesis in unconditioned vs TIS108. (b) Pathway of gibberellic acid biosynthesis in unconditioned vs GR24. (c) Pathway of abscisic acid biosynthesis in unconditioned vs conditioned. (d) Pathway of ethylene biosynthesis in unconditioned vs FL+GA_3_.**


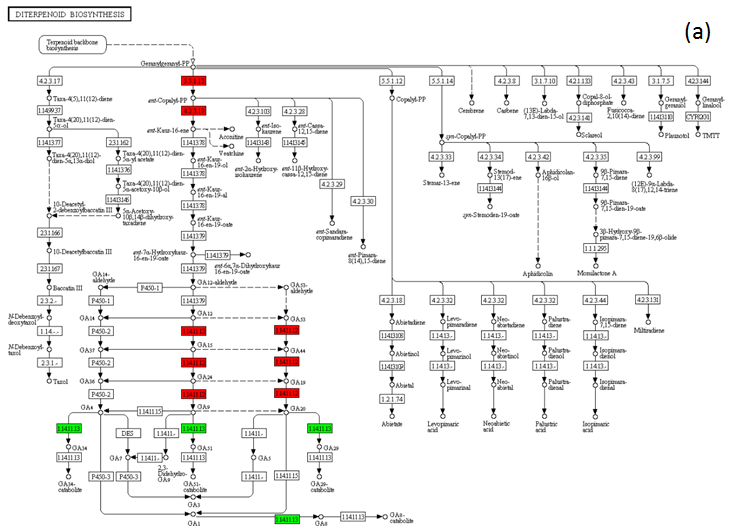

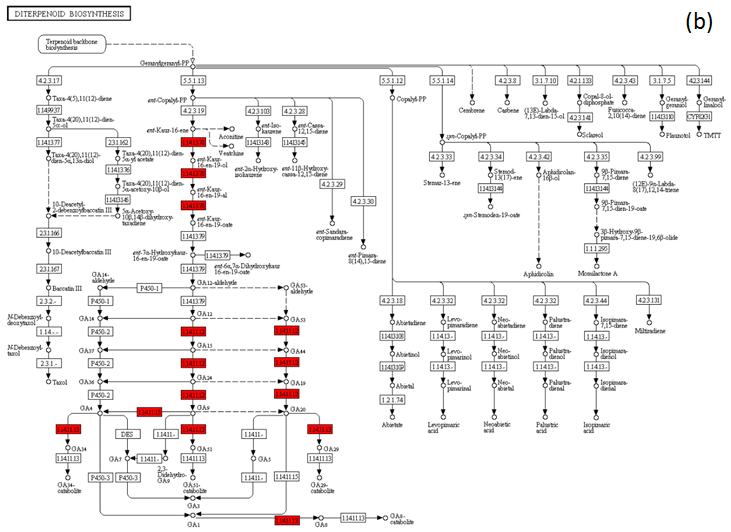


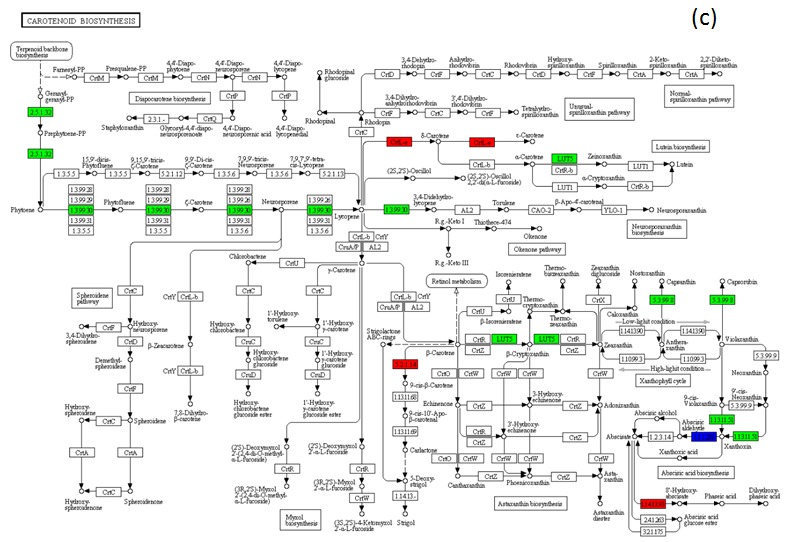

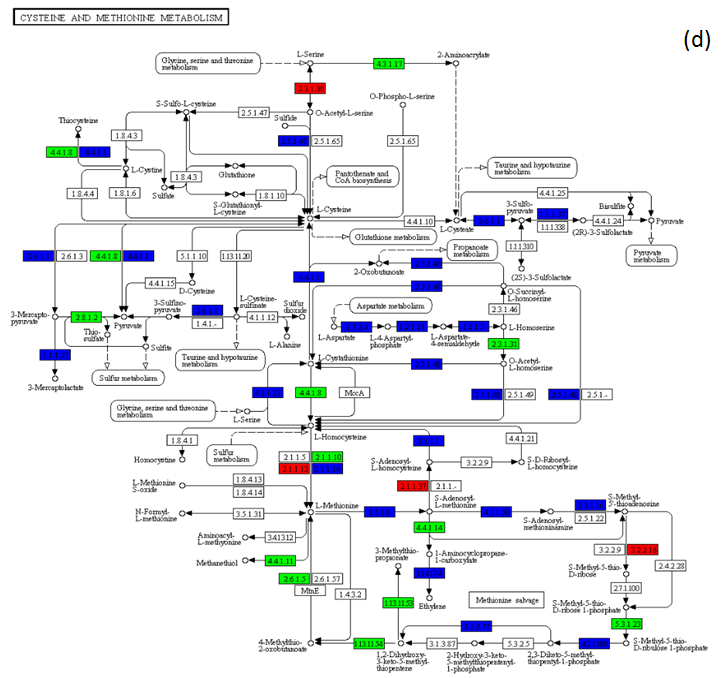

Supplement: S1 Fig — (a) Pathway of gibberellic acid biosynthesis in unconditioned vs TIS108. (b) Pathway of gibberellic acid biosynthesis in unconditioned vs GR24. (c) Pathway of abscisic acid biosynthesis in unconditioned vs conditioned. (d) Pathway of ethylene biosynthesis in unconditioned vs FL+GA3. (DOCX) [file pone.0187539.s008.docx]
