# Supplementary figures and images for: Transcriptome analysis of Phelipanche aegyptiaca seed germination mechanisms stimulated by fluridone, TIS108, and GR24
Source: PLoS One. 2017 Nov 3;12(11):e0187539. doi: 10.1371/journal.pone.0187539 (PMC5669479; doi:10.1371/journal.pone.0187539)

S2 Fig. Species distribution annotated in the NR database


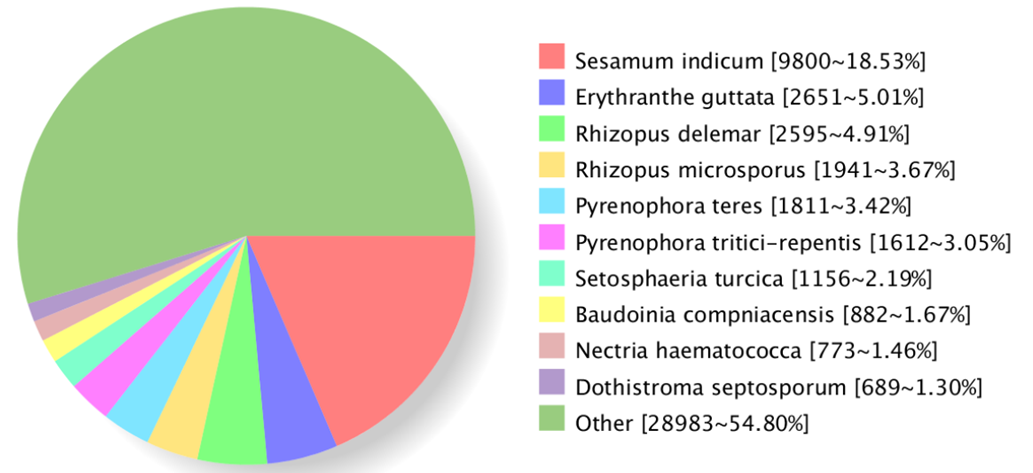

Supplement: S2 Fig — (DOCX) [file pone.0187539.s009.docx]
